# The impact and cost-effectiveness of introducing the 10-valent pneumococcal conjugate vaccine into the paediatric immunisation programme in Iceland—A population-based time series analysis

**DOI:** 10.1371/journal.pone.0249497

**Published:** 2021-04-08

**Authors:** Elias Eythorsson, Tinna L. Ásgeirsdóttir, Helga Erlendsdóttir, Birgir Hrafnkelsson, Karl G. Kristinsson, Ásgeir Haraldsson

**Affiliations:** 1 Faculty of Medicine, University of Iceland, Reykjavik, Iceland; 2 Faculty of Economics, University of Iceland, Reykjavik, Iceland; 3 Department of Clinical Microbiology, Landspitali–The National University Hospital of Iceland, Reykjavik, Iceland; 4 Department of Mathematics, University of Iceland, Reykjavik, Iceland; 5 Children’s Hospital Iceland, Landspitali–The National University Hospital of Iceland, Reykjavik, Iceland; National Center for Global Health and Medicine, JAPAN

## Abstract

**Introduction:**

*Streptococcus pneumoniae* is a cause of infections that range in severity from acute otitis media (AOM) to pneumonia and invasive pneumococcal disease (IPD). The 10-valent pneumococcal conjugate vaccine (PHiD-CV10) was introduced into the Icelandic paediatric immunisation programme in 2011. The aim was to estimate the population impact and cost-effectiveness of PHiD-CV10 introduction.

**Methods:**

Data on primary care visits from 2005–2015 and hospitalisations from 2005–2017 were obtained from population-based registries. A Bayesian time series analysis with synthetic controls was employed to estimate the number of cases of AOM, pneumonia and IPD that would have occurred between 2013–2017, had PHiD-CV10 not been introduced. Prevented cases were calculated by subtracting the observed number of cases from this estimate. The cost of the programme was calculated accounting for cost-savings due to prevented cases.

**Results:**

The introduction of PHiD-CV10 prevented 13,767 (95% credible interval [CI] 2,511–29,410) visits for AOM from 2013–2015, and prevented 1,814 (95%CI -523-4,512) hospitalisations for pneumonia and 53 (95%CI -17-177) admissions for IPD from 2013–2017. Visits for AOM decreased both among young children and among children 4–19 years of age, with rate ratios between 0.72–0.89. Decreases were observed in both pneumonia hospitalisations (rate ratios between 0.67–0.92) and IPD (rate ratios between 0.27–0.94). The total cost of implementing PHiD-CV10 in Iceland was -7,463,176 United States Dollars (USD) (95%CI -16,159,551–582,135) with 2.1 USD (95%CI 0.2–4.7) saved for every 1 USD spent.

**Conclusions:**

The introduction of PHiD-CV10 was associated with large decreases in visits and hospitalisations for infections commonly caused by pneumococcus and was cost-saving during the first five years of the immunisation programme.

## Introduction

Pneumococcal conjugate vaccines (PCV) have been shown to provide direct protection against acute otitis media (AOM), pneumonia and invasive pneumococcal disease (IPD) in randomised controlled trials [1–4]. Vaccines can also provide indirect protection among unvaccinated individuals through herd immunity. Indirect protection against IPD and pneumonia have been reported in unvaccinated children and adults [5–7]. However, indirect protection against AOM is less established [8, 9].

The combined direct and indirect effects of PCV are generally estimated by comparing rates of infection after vaccine introduction to pre-vaccine rates in order to infer the total impact of vaccination. This approach is unable to separate the effects of vaccine introduction from coincident changes in healthcare utilization and reporting unrelated to the vaccine. Methods to mitigate this that utilise time series analysis and trends in unrelated diseases as synthetic controls have been developed and have been used to estimate the impact of PCV on pneumonia hospitalisations and mortality [6, 10–12].

Cost-effectiveness analyses have shown PCV to be a cost-effective intervention [13]. However, most of the studies were conducted before the vaccine was implemented, and the results depend on assumptions rather than observations with regard to both direct and indirect protection [14]. Two post-implementation cost-effectiveness studies of PCV have been published, which evaluated the introduction of the 7-valent PCV in the United States in 2000 and in Australia in 2005 [15, 16]. Neither study directly estimated the impact of PCV on AOM, pneumonia or IPD but instead relied on previously published estimates or efficacy data. Both found that cost-effectiveness was dependent on indirect protection.

In 2011, the 10-valent pneumococcal *Haemophilus influenzae* Protein D conjugate vaccine (PHiD-CV, Synflorix®) was incorporated into the Icelandic paediatric immunisation programme, with a two plus one schedule given at three, five and 12 months of age. Vaccine uptake was immediately high with over 97% of vaccine eligible children receiving the primary vaccination by their first birthday [17]. Vaccine eligible birth cohorts (2011–2015) in Iceland have previously been shown to have experienced fewer AOM visits and hospitalisations for pneumonia and IPD compared to vaccine non-eligible birth cohorts (2005–2010) [9, 18].

The aim of this study was to evaluate the direct and indirect impact of the vaccinations on AOM, pneumonia and IPD in Iceland, and analyse post-implementation cost-effectiveness using directly observed data.

## Materials and methods

### Data sources

The study is a population-based time series analysis. Data were obtained from the Primary Care Registry and National Drug Prescription Registry of the Icelandic Directorate of Health, and the patient registry of Landspitali–The National University Hospital of Iceland. Data were linked between registries using national identification numbers, and subsequently anonymised before being released to the study’s authors. During data extraction, International Classification of Diseases, 10th revision (ICD-10) diagnostic codes were not restricted to the primary diagnosis and all codes associated with each visit or hospitalisation were included. Multiple visits or hospitalisations by the same individual associated with the same ICD-10 code within the same calendar-month were grouped together. No data had missing identification numbers. The study was approved by The National Bioethics Committee (VSNb2013010015/03.07), the National Data Protection Authority (2013010100VEL/—) and the Directorate of Health, Iceland (1301266/5.6.1/gkg)

The Primary Care Registry contains information on all primary care visits in Iceland. Visits associated with diagnostic codes compatible with AOM were extracted and used as the outcome measure. Visits associated with other chapters of the ICD-10 diagnostic coding system were also extracted as synthetic controls (Table 1). The observation period was 1 January 2005 to 31 December 2015, as the Primary Care Registry was not updated for 2016 and 2017. Data on all outpatient antimicrobial prescriptions were extracted from the National Drug Prescription Registry using Anatomical Therapeutic Chemical code J01. AOM visits associated with an antimicrobial prescription were used as an alternate case-definition in sensitivity analyses.

**Table 1:** The International Classification of Diseases, 10th revision (ICD-10) codes used to define disease states and synthetic controls used in the time series analyses.

Group	ICD-10	Description	Exclusions
Outcomes and alternatives	H65, H66, H70, H72	Acute otitis media	-
-	H65, H66, H70, H72, and antimicrobial prescription	Acute otitis media (alternate)	-
-	H66	Acute otitis media (alternate)	-
-	J12, J13, J14, J15, J16, J17, J18	Pneumonia	-
-	J13, J15.8, J15.9, J18.1, J18.8, J18.9	Pneumonia (alternate)	-
-	J13, J15.8, J15.9, J18.1, J18.8, J18.9, and microbiological and radiographical testing	Pneumonia (alternate)	-
-	Any or none	Positive pneumococcal culture or PCR from normally sterile site	-
-	Any or none	Positive vaccine-type culture or PCR from normally sterile site (alternate)	-
-	N10, N30.0, N39.0	Urinary tract infection (alternate)	-
ICD-10 chapters	A10-B99	Certain infectious and parasitic diseases	A40.3, B95
-	C00-D48	Neoplasms	-
-	D50-89	Diseases of the blood and blood-forming organs and certain disorders involving the immune mechanism	-
-	E00-99	Endocrine, nutritional and metabolic diseases	-
-	G00-G99	Diseases of the nervous system	G00-G04
-	H00-99	Diseases of the eye and adnexa, Diseases of the ear and mastoid process	H10, H65, H66
-	I00-99	Diseases of the circulatory system	-
-	K00-99	Diseases of the digestive system	-
-	L00-99	Diseases of the skin and subcutaneous tissue	-
-	M00-99	Diseases of the musculoskeletal system and connective tissue	-
-	N00-99	Diseases of the genitourinary system	-
-	P00-99	Certain conditions originating in the perinatal period	-
-	Q00-99	Congenital malformations, deformations and chromosomal abnormalities	-
-	R00-99	Symptoms, signs and abnormal clinical and laboratory findings, not elsewhere classified	-
-	S00-T99	Provisional assignment of new diseases of uncertain etiology	-
-	U00-99	Injury, poisoning and certain other consequences of external causes	-
-	V00-Y99	External causes of morbidity	-
-	Z00-99	Factors influencing health status and contact with health services	-
Other grouped and specific outcomes	J20-22	Bronchitis, bronchiolitis and unspecified acute lower respiratory infection	-
-	B20-24	HIV	-
-	E10-14	Diabetes	-
-	I60-64	Stroke	-
-	A09, K52.9, K59.1, R19.7	Gastroenteritis and Diarrhea	-
-	P05-07	Premature delivery and low birth weight	-
-	K35	Appendicitis	-
-	K80	Cholelithiasis	-
-	E86	Dehydration	-
-	A00-Z99	All non-respiratory visits or hospitalizations	J00-J99, F and O chapters

Data for patients hospitalised for pneumonia and IPD were obtained from Landspitali University Hospital’s patient registry for the period 2005–2017. Hospitalised pneumonia was defined as any hospital admission associated with ICD-10 diagnostic codes compatible with pneumonia (Table 1). Hospitalised IPD was based on microbiological data from the Department of Clinical Microbiology at Landspitali University Hospital and linked to the patient registry. Hospitalised IPD was defined as any hospital admission associated with culture or PCR-confirmed *Streptococcus pneumoniae* from joint fluid, bone, cerebrospinal fluid or blood, regardless of ICD-10 discharge diagnosis. Hospitalisations associated with specific chapters of the ICD-10 coding system were extracted and used as synthetic controls (Table 1).

AOM, pneumonia and IPD were used as outcome measures. Each was given two alternate case-definitions that were used in sensitivity analyses, along with urinary tract infection as a negative control. Outpatient antimicrobial prescriptions were obtained from the National Drug Prescription Registry and linked to visits using national identification numbers. Inpatient microbiological and radiographical testing was ascertained through cost data. All other codes were used as synthetic controls. When applicable, codes associated with pneumococcal infections were excluded from control groupings.

For each hospitalisation or visit to the emergency department, a detailed breakdown of associated costs was available, and was extracted for each of the disease categories included in the study. No cost data were available for primary care visits. Because the Children’s Hospital’s paediatric emergency department serves as a walk-in clinic for the greater capital area, the distribution of costs for otitis media visits to the emergency department was assumed to reflect that of primary care visits. The number of PHiD-CV10 doses purchased by the Government and the unit price for each dose per calendar year were obtained from the Directorate of Health. The yearly employment rate of individuals 15 to 24, 25 to 54, and 55 to 64 years old, from 2011–2017, was extracted from The Organization for Economic Cooperation and Development (OECD) Labour Force Statistics [19], and the deciles of regular total wage for working Icelanders from 2011–2017 obtained from Statistics Iceland (www.statice.is). The consumer price index for medical care obtained from Statistics Iceland was used to convert all direct health care costs to 2015 price levels in Icelandic kronas (www.statice.is). All costs were converted to United States Dollars (USD) using the official exchange rates of the Icelandic Central Bank (www.sedlabanki.is).

### Impact of PHiD-CV10

The impact of PHiD-CV10 introduction on the incidence of pneumococcal disease was calculated using Bayesian time series methods [6, 20] and the result used as an input for a cost-effectiveness analysis. The pre-vaccine period was defined as 1 January 2005 to 31 December 2010, and the post-vaccine period as 1 January 2013 to 31 December 2017. The transition period during which neither baseline trends nor vaccine impact were estimated was defined as 1 January 2011 to 31 December 2012, which spans the period during which the first vaccine eligible birth cohort 2011 started and finished its vaccination schedule. For each disease category and age-group, four models of PHiD-CV10 impact were estimated; an interrupted time series (ITS) with all non-respiratory visits or hospitalisations as an offset, and ITS without offset, a principal component analysis [20], and a synthetic control model [6]. All were Bayesian Poisson models with observation-specific random intercepts to account for over-dispersion. Each model utilized the pre-vaccine period to predict the monthly occurrence of the outcome of interest in the post-vaccine period, assuming the vaccination had not occurred. These models were then combined using a Bayesian model-stacking procedure [21]. The methods are described further in the S1 Appendix.

From the posterior predictive distribution of the stacked model, a total of 10,000 Markov chain Monte Carlo (MCMC) samples were drawn. These represented the number of cases that would have occurred in the post-vaccine period, had the vaccine not been introduced. The first 2,000 MCMC draws were discarded for optimal burn-in. For each of the remaining 8,000 draws, the rate ratio (*RR*) between the observed and predicted number of cases during the post-vaccine period was calculated, and the median and 95% credible intervals (CI) extracted from the resulting distribution of rate ratios. Vaccine impact was defined as 1-*RR*. To estimate the onset of vaccine impact, the rate ratio was calculated over a rolling 12-month period, the first of which included 11-months of pre-vaccine data and one month of post-vaccine data. The number of cases prevented by the vaccine was calculated for each calendar-month, by subtracting the observed number of cases from each of the 8,000 MCMC draws. Finally, the cumulative sum of prevented cases was calculated, and the median and 95% credible intervals extracted.

### Cost-effectiveness analysis

The cost-effectiveness of PHiD-CV10 introduction as compared to no intervention was estimated from both the healthcare and societal perspectives. The societal perspective included both direct and indirect costs associated with productivity loss, while analysis from the healthcare perspective included only direct costs. Neither analysis included estimates of long-term sequelae or their associated costs. The time horizon was five years and both costs and cost-savings were discounted at a 3% discount rate. All costs were presented in 2015 USD. A summary of the cost-effectiveness parameters and assumptions are presented in Table 2.

**Table 2:** A summary of the parameters and assumptions of the cost-effectiveness analysis.

Parameter or Assumption	Description	Distribution
Perspective	Healthcare, Societal	-
Comparators	PHiD-CV10 vs no vaccine	-
Model	Ecological time series	-
Time horizon	5 years	-
Price date	2015 USD	-
Discount rate	3%	~ Triangle (0, 6)
Cost of vaccine	Directly observed	~ N (observed, 5 USD)
Cost of visit or hospitalization	Directly observed	Resampling with replacement
Wage	Observed	~ logN (12.85, 0.35)
Employment	Observed	~ Binomial (observed)
Days of work lost	Estimated from observed hospital length of stay	Resampling with replacement from hospital length of stay + ~Pois (1/2 hospital length of stay)
Vaccine uptake	Implicitly included	-
Vaccine coverage	Implicitly included	-
Serotype replacement	Implicitly included	-
Herd-effect	Implicitly included	-

All costs were first adjusted to constant 2015 values and converted to USD. The cost of the vaccine, visits and hospitalisations were directly observed. The possible vaccine costs were assumed to follow a normal distribution with mean equal to the observed costs, and the standard deviation equal to five USD. The distribution of costs associated with visits and hospitalisations was empirically estimated using resampling with replacement. Wage and employment rates were obtained from Statistics Iceland and wage was optimally fitted using a lognormal distribution.

The direct cumulative savings associated with PHiD-CV10 introduction were calculated by multiplying the predicted number of prevented cases from the Bayesian time series analysis with the expected cost of each case. The expected cost was obtained through sampling with replacement from the observed costs extracted from Landspitali University Hospital’s patient registry. The sampling was stratified by disease category and age-group. The direct costs associated with the introduction of PHiD-CV10 into the paediatric immunisation programme were obtained from the Directorate of Health. Wastage was accounted for, as costs included doses never administered. However, additional administration costs were not included, as each dose was administered during the same visit as other established vaccines. The direct costs associated with the vaccine were subtracted from the direct cumulative savings to obtain the final estimate of the total cost. This resulted in 8,000 posterior draws of the total cost, from which the median and 95% credible intervals were extracted.

The societal perspective included indirect costs due to productivity loss. The deciles of wage extracted from Statistics Iceland were optimally fitted to a lognormal distribution to obtain a continuous wage distribution. Days of lost work were assumed. For each case of otitis media in primary care, days of work lost by a parent or guardian were assumed to follow a Poisson distribution with mean equaling one. For each pneumonia or IPD hospitalisation, days of work lost were assumed to equal 1.5 times the duration of the hospital stay, with hospital length of stay sampled with replacement from the observed data obtained from the patient registry. The indirect costs were calculated by multiplying the days of work lost with wages sampled from the lognormal wage distribution, accounting for the employment rate. Cost-effectiveness was summarised with incremental cost-effectiveness ratios (ICER) with 95% CI. Return on investment was calculated as the cost-savings minus the cost expressed as a proportion of the cost.

### Sensitivity analysis

Three sensitivity analyses were performed to validate the robustness of the impact models, as well as a falsification test. Firstly, the sensitivity of the impact models to the choice of pre-vaccine period was evaluated by selecting different pre-vaccine periods (2005–2007, 2005–2008, 2005–2009, and 2005–2010) and refitting the models for each disease category and age-group. Secondly, the robustness of the synthetic control models with regards to the included covariates, was evaluated by removing the top covariate and re-estimating the rate ratio. This was done iteratively three times and the resulting three rate ratios compared to the one obtained in the main analysis. Thirdly, several different case-definitions for each disease category were explored. The models were refitted with two additional definitions of otitis media (visits with ICD-10 code H66 only, and visits with ICD-10 codes H65, H66, H70, H72, associated with a filled antimicrobial prescription), and two additional definitions of pneumonia (hospitalisations with ICD-10 codes J13, J15.8, J15.9, J18.1, J18.8 and J18.9; and hospitalisations with ICD-10 codes J13, J15.8, J15.9, J18.1, J18.8 and J18.9 in which microbiological and radiological examinations had been performed). The impact of PHiD-CV10 on vaccine-type IPD was also evaluated. Finally, the models were re-fitted with urinary tract infections as the outcome variable, to evaluate the specificity of the results regarding infections likely to be caused by pneumococci. The cost-effectiveness analysis included a built in probabilistic sensitivity analysis in which all assumptions were simultaneously varied over their respective probability distributions (Table 2). The results of the sensitivity analysis were included in all reported credible intervals.

All data, statistical code and raw results are accessible at https://osf.io/u9g65/.

## Results

### Population impact on acute otitis media in children younger than 20 years of age

**Fig 1:**
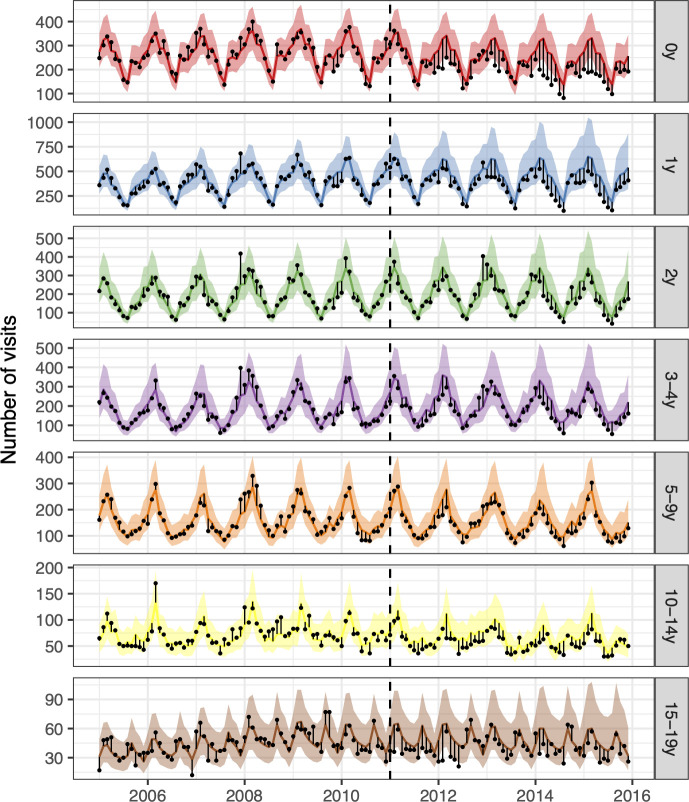
The observed and predicted number of AOM visits from 1 January 2005 to 31 December 2015 for each age-group. Observed visits are illustrated as black points, the posterior predicted visits are presented as lines and 95% credible intervals as a shaded area. The start of the vaccine period is delineated with a vertical black dashed line. The distance between the observed and predicted visits for each calendar-month is depicted by a thin black line. Assuming that the model was correct, and that no intervention took place, the black points would have an equal probability of occurring above or below the prediction line. Points below the lower bound of the shaded area would then represent observations with less than a 2.5% probability of occurring. The scale of the Y-axes differs between age-groups.

From 1 January 2005 to 31 December 2015, children younger than 20 years of age visited primary care physicians 164,453 times for AOM or its complications. Both AOM and overall visits decreased in the post-vaccine period in all age-groups. (S1 Fig). With few exceptions, observed AOM visits were fewer than predicted in the post-vaccine period (Fig 1). The posterior predictions of each component model are shown in S2 Fig, and the weights used to produce the final stacked model are presented in S1 Table.

**Fig 2:**
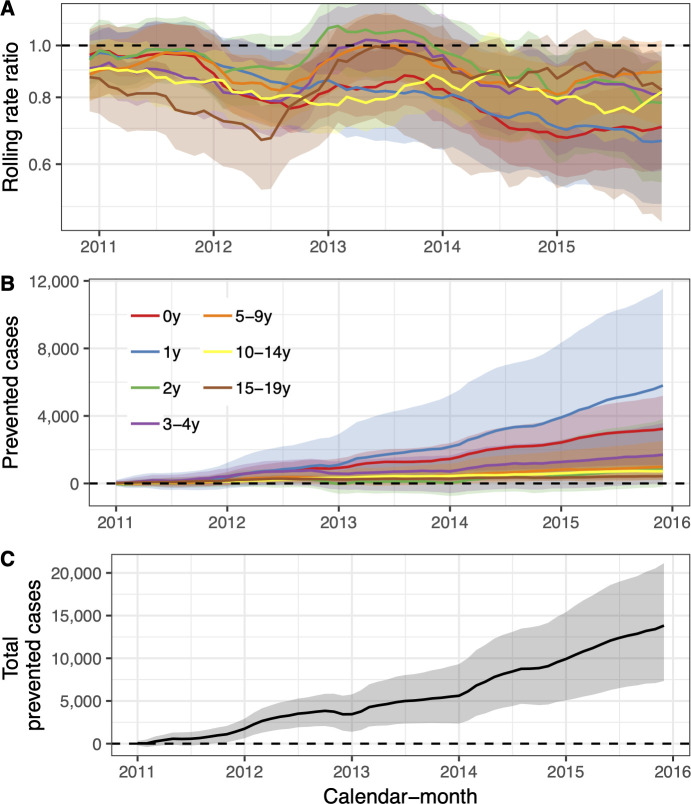
The impact of the vaccine (PHiD-CV10) on acute otitis media (AOM) and complications. In Panel A, the estimated 12-month rolling rate ratio between observed and predicted AOM cases is shown per age-group, and the 95% credible intervals (CI) are illustrated as a shaded area. Panel B depicts the cumulative number of prevented AOM cases during the post-vaccine period (2011–2015) for each age-group, along with 95% CI. The total cumulative prevented AOM cases regardless of age-group is shown in Panel C.

The 95% credible interval of the rate ratio between the observed and the predicted number of AOM visits was less than one in all age-groups, indicating a 97.5% or greater probability that the rate of AOM decreased due to the introduction of PHiD-CV10 (Fig 2, Table 3). The decrease was largest among young children; 26% (12%-36%) in children younger than one year of age and 28% (5%-42%) in children one year of age. In total, PHiD-CV10 prevented 13,767 (95% credible interval 2,511 to 29,410) cases of AOM between 2013–2015. The largest decreases in the cumulative number of prevented cases were seen in the youngest age-groups (Fig 2, Table 3). The result was invariant to different case-definitions, the inclusion of different controls and different pre-vaccine periods (S3–S5 Figs). Additionally, the falsification test was negative as the model did not predict a decrease in urinary tract infections (S6 Fig)

**Table 3:** The rate ratio between observed and predicted primary care visits due to Acute Otitis Media (AOM) and complications during the post-vaccine period (2013–2015).

Age-group	Rate ratio (95% CI)	Cumulative cases prevented (95% CI)	Direct savings (USD, 95% CI)	Indirect savings (USD, 95% CI)
0y	0.74 (0.64–0.88)	3,234 (1,008 to 5,195)	305,330 (90,933 to 514,848)	45,386 (11,143 to 84,654)
1y	0.72 (0.58–0.95)	5,802 (817 to 11,526)	530,468 (57,564 to 1,150,759)	74,298 (3,778 to 193,180)
2y	0.88 (0.66–0.98)	900 (-185 to 3,817)	92,117 (-52,649 to 407,227)	14,377 (-11,004 to 64,562)
3-4y	0.86 (0.69–0.97)	1,702 (21 to 3,576)	135,274 (-16,985 to 357,905)	23,880 (-4,324 to 62,811)
5-9y	0.88 (0.73–0.96)	979 (229 to 2,521)	134,548 (-38,612 to 430,729)	14,242 (-1,030 to 40,961)
10-14y	0.83 (0.75–0.92)	720 (411 to 1,086)	113,333 (4,669 to 285,816)	10,313 (-3,098 to 20,035)
15-19y	0.89 (0.56–0.98)	430 (210 to 1,689)	55,819 (-8,278 to 227,493	6,169 (698 to 25,248)

Estimates are presented with 95% credible intervals (95% CI) for the seven age-groups included in the study. The predicted cumulative number of prevented cases as of 1 December 2015 is also presented. A negative number indicates that there is a non-zero probability that the vaccine caused more AOM visits to occur. Direct and indirect savings are presented in 2015 USD.

### Population impact on pneumonia hospitalisations

In total, 13,373 pneumonia hospitalisations occurred between 1 January 2005 to 31 December 2017. Observed trends in pneumonia hospitalisations during the study period are shown in S7 Fig.

**Fig 3:**
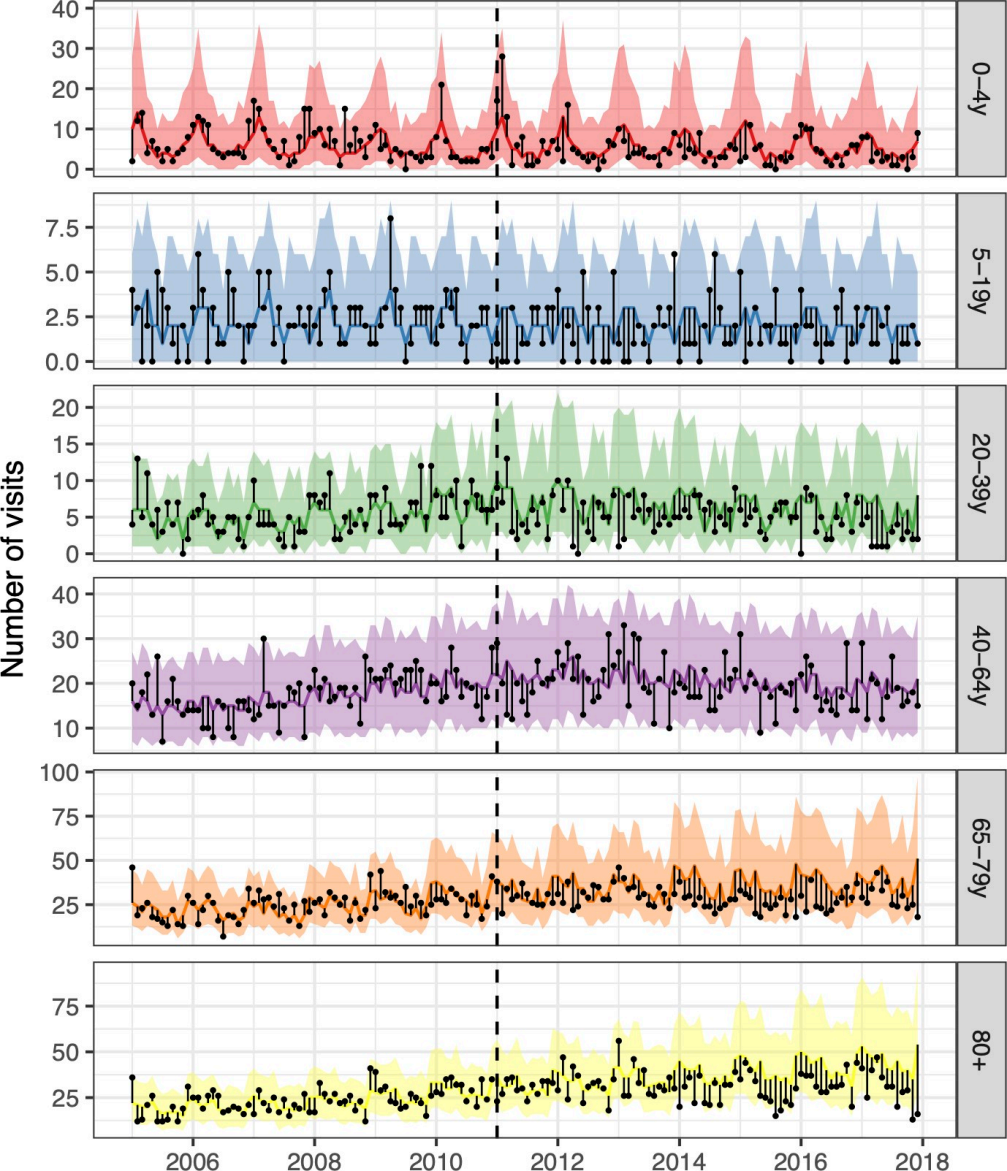
The observed and predicted number of pneumonia hospitalisations from 1 January 2005 to 31 December 2017 for each age-group. Observed cases are illustrated as black points. The predicted number of hospitalisations are presented as lines and 95% credible intervals as shaded areas. The start of the vaccine period is delineated with a vertical black dashed line. The distance between the observed and the predicted hospitalisations for each calendar-month is depicted by a thin black line. Assuming that the model was correct, and no intervention occurred, the black points would have an equal probability of appearing above or below the prediction line. The scale of the Y-axes differs between age-groups.

During most of the post-vaccine period, the observed number of pneumonia hospitalisations was equal to or below the prediction line among children zero to four years of age, and among adults 20 to 39, 65 to 79, and 80 years of age and older (Fig 3). The posterior predictions of the component models are shown in S8 Fig and the weights used to produce the final stacked model are presented in S1 Table.

**Fig 4:**
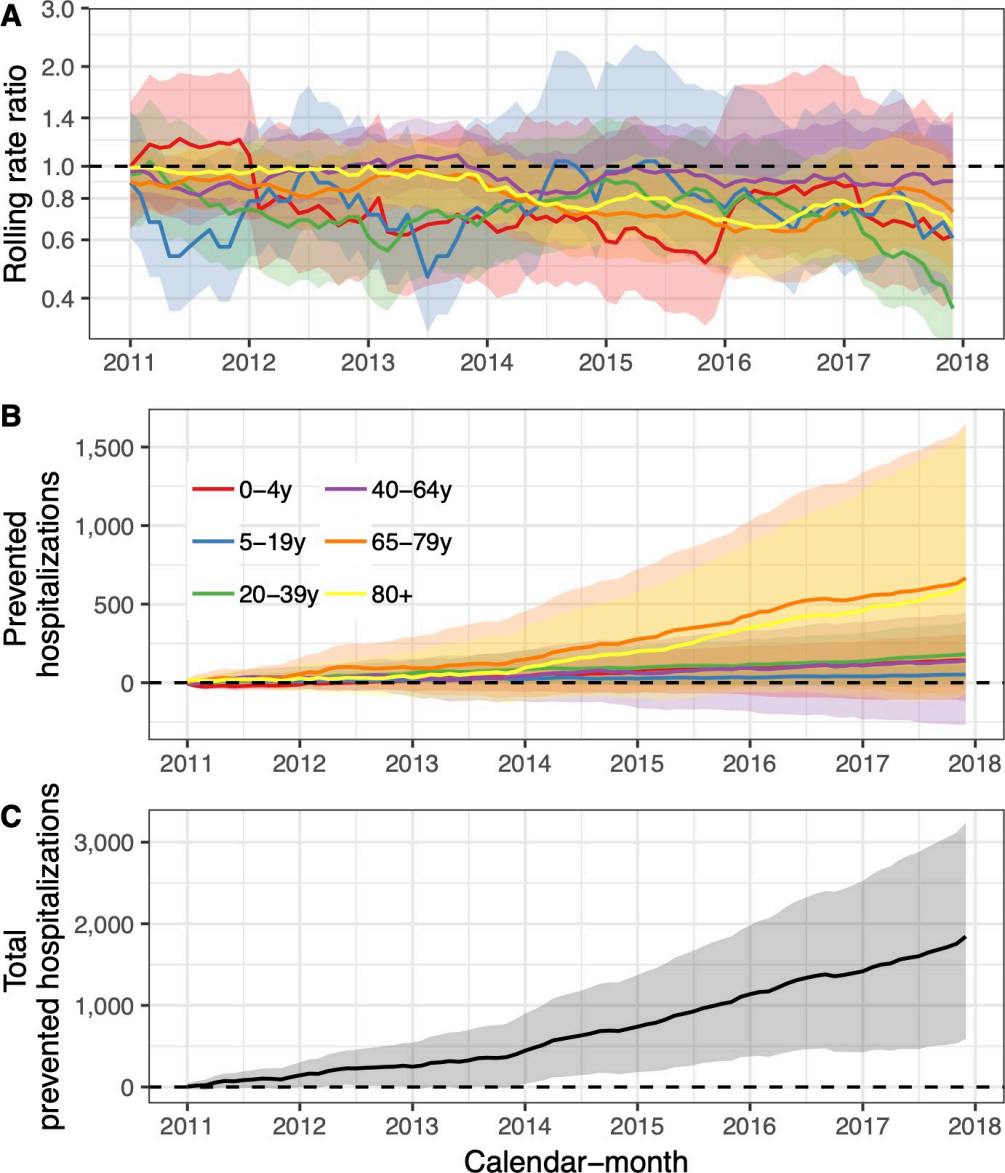
The population impact of the vaccine (PHiD-CV10) on pneumonia hospitalisations is summarized. In Panel A, the estimated 12-month rolling rate ratio between observed and predicted pneumonia hospitalisations is shown per age-group, and the 95% credible intervals (CI) are illustrated as a shaded areas. Panel B depicts the cumulative number of prevented pneumonia hospitalisations during the post-vaccine period (2011–2017) for each age-group along with 95% CI. The total cumulative prevented pneumonia hospitalisations regardless of age-group is shown in Panel C.

Among children zero to four years of age, the posterior median of the rate ratio was 0.67. Though the 97.5% credible limit was above the threshold value of one, there was a 94% probability that the rate ratio was lower than one, and a 90% probability that it was lower than 0.83. Similarly, the posterior median of the rate ratio was 0.74 among children five to 19 years of age, and there was a 90% probability that the rate ratio was lower than one. Among adults 65 to 79 years of age and 80 years of age and older, the posterior median of the rate ratio was 0.75 and 0.76 respectively, and both had a 97% probability of being lower than one. In total, the introduction of PHiD-CV10 prevented 1,814 (95% credible interval -523 to 4,512) hospital admissions for pneumonia from 2013–2017 (Fig 4, Table 4).

**Table 4:** The posterior median of the rate ratio between observed and predicted number pneumonia hospitalisations during the post-vaccine period (2013–2017).

Age-group	Rate ratio (95% CI)	Cumulative cases prevented (95% CI)	Direct savings (USD, 95% CI)	Indirect savings (USD, 95% CI)
0-4y	0.67 (0.51–1.39)	142 (-115 to 307)	444,533 (-44,181 to 1,309,917)	52,535 (-59,043 to 136,715)
5-19y	0.74 (0.54–1.35)	52 (-27 to 113)	234,848 (-236,236 to 748,522)	20,472 (-18,876 to 61,481)
20-39y	0.68 (0.51–0.95)	182 (14 to 384)	968,662 (-203,048 to 2,567,059)	70,071 (-9,442 to 164,747)
40-64y	0.92 (0.79–1.22)	141 (-270 to 445)	933,290 (-2,748,49 to 4,848,557)	71,953 (-113,414 to 223,171)
65-79y	0.75 (0.55–1.02)	666 (-49 to 1,648)	5,476,585 (-910,021 to 15,590,280)	323,964 (-4,745 to 786,252)
80+	0.76 (0.56–1.02)	631 (-76 to 1,615)	4,664,256 (-817,266 to 13,013,699)	287,270 (-37,961 to 742,168)

The rate ratio is presented with 95% credible intervals (95% CI) for the six age-groups included in the study. The predicted cumulative number of prevented cases as of 1 December 2017 is also presented. A negative number indicates a non-zero probability that the vaccine caused more pneumonia hospitalisations to occur. Direct and indirect savings are presented in constant 2015 USD.

Sensitivity analysis found that stricter definitions of pneumonia resulted in significantly greater predicted declines than the base-case analysis (S9 Fig). The inclusion of different synthetic controls did not change the overall result (S10 Fig). Some component models became unstable when only three years (2005–2007) of pre-vaccine data were used to fit them (S11 Fig). The falsification test was negative, as the model did not predict a decrease in hospitalisations for urinary tract infections (S12 Fig).

### Population impact on hospital admissions for invasive pneumococcal disease

From 1 January 2005 to 31 December 2016, there were 338 hospitalisations for culture-confirmed IPD among all age-groups. Of these, 206 occurred before the introduction of PHiD-CV10 into the paediatric immunisation programme in Iceland. In all age-groups, standardised hospitalisations for IPD decreased relative to standardised hospital admissions regardless of cause (S13 Fig).

**Fig 5:**
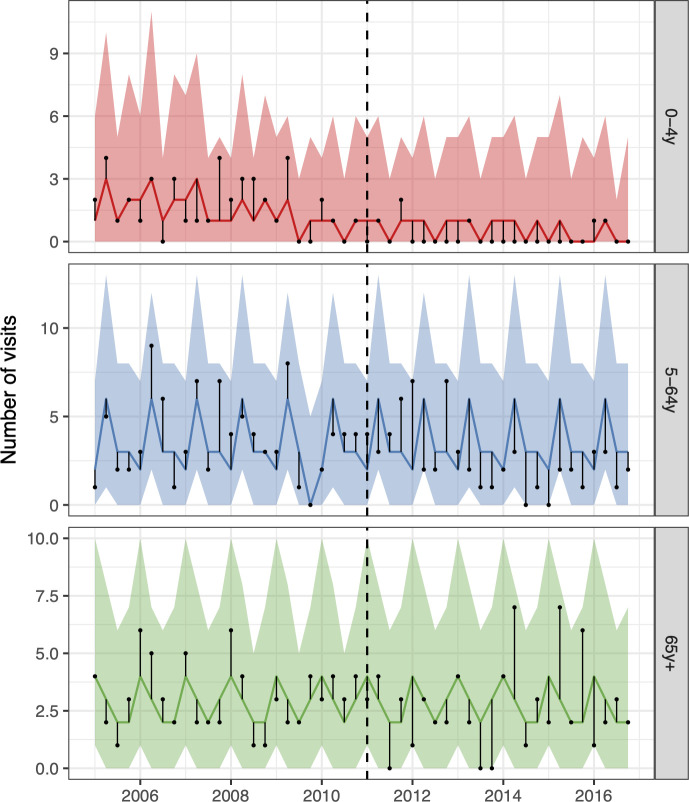
The observed and predicted number of IPD hospitalizations from 1 January 2005 to 31 December 2016 for each age-group. Observed cases are illustrated as black points, and the predicted number of cases are presented as lines with 95% credible intervals as a shaded area. The start of the vaccine period is shown with a vertical black dashed line. The distance between the observed and predicted cases for each year-quarter is depicted by a thin black line. Assuming that the model was correct, and no intervention took place, the black points would have an equal probability of occurring above or below the prediction line.

Among children zero to four years of age, observed IPD hospitalisations were equal to or fewer than the predicted hospitalisations in all but two quarters (Fig 5). Similarly, observed hospitalisations among individuals five to 64 years of age were fewer than predicted. The posterior predictions of the component models are shown in S14 Fig and the stacking weights are shown in S1 Table.

**Fig 6:**
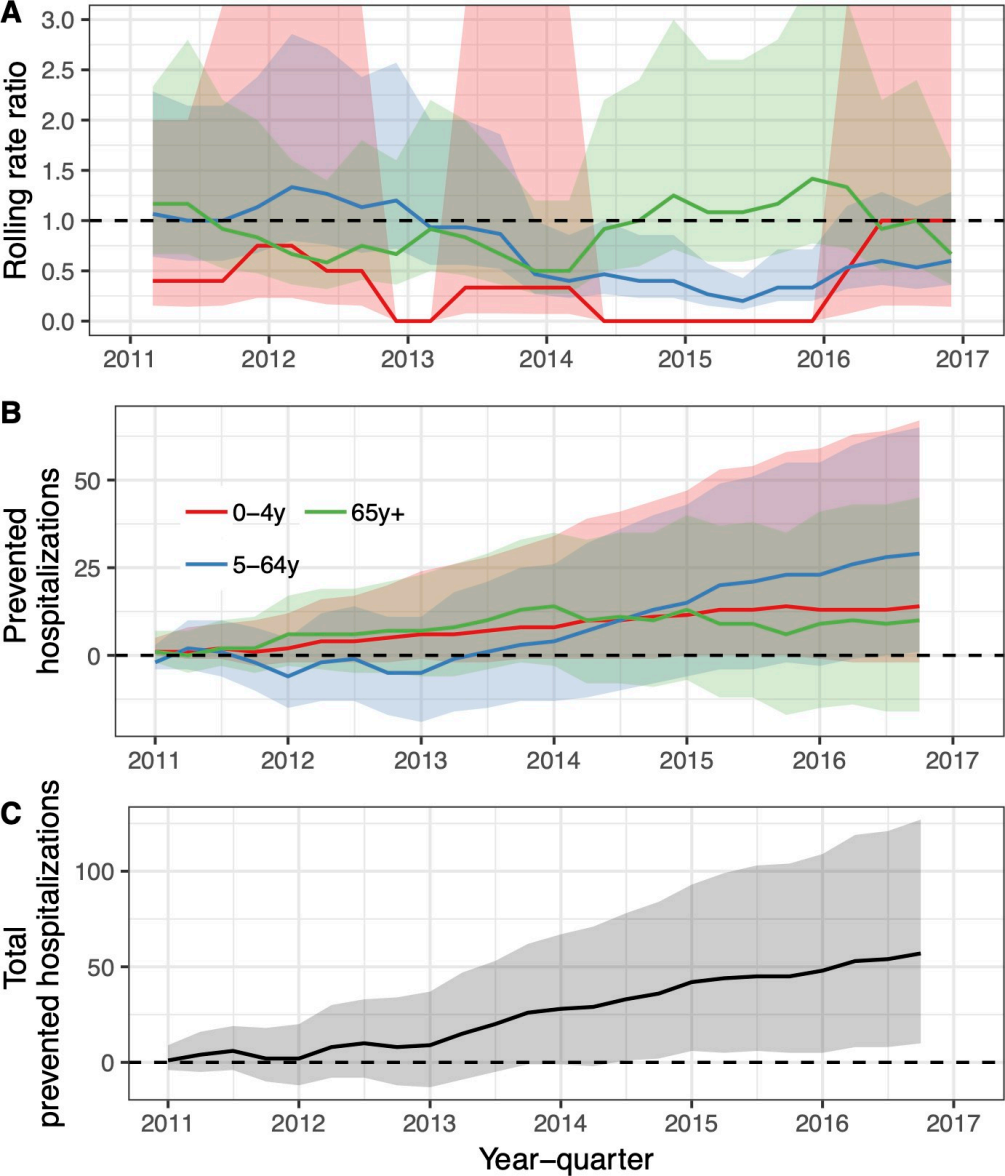
The population impact of the vaccine (PHiD-CV10) on hospital admissions for invasive pneumococcal disease. In Panel A, the estimated 12-month rolling rate ratio between the observed and the predicted number of IPD hospitalisations in the post-vaccine period (2011–2016) is shown per age-group. In some 12-month periods, no IPD hospitalisations were observed, and the resulting rate ratio was zero regardless of the denominator. In other periods, 2.5% or more of the MCMC draws predicted zero IPD hospitalisations, which resulted in a 95% credible intervals of the rate ratio that extended towards infinity. These issues do not change the overall interpretation of the prediction line presented. Panel B depicts the cumulative number of prevented IPD hospitalisations during the post-vaccine period (2011–2015) for each age-group.

The posterior median of the rate ratio for children younger than five years of age was 0.27, corresponding to a 50% probability that the vaccine impact was greater than or equal to 73% (Fig 6, Table 5). The 95% credible intervals of the rate ratio were wide, reflecting uncertainty due to the few number of IPD hospitalisations. However, 90% of the MCMC draws of the rate ratio were below 0.75 and 93% were under the threshold value of one. The 95% credible interval of the rate ratio among individuals five to 64 years of age was lower than one, indicating a 97.5% or greater probability that the rate of IPD hospitalisation decreased in this age-group following the introduction of PHiD-CV10. In total, the introduction of PHiD-CV10 prevented 53 (95% credible interval -17 to 177) hospitalisations for IPD between 2013–2016.

**Table 5:** The rate ratio between observed and predicted number of hospital admissions for Invasive Pneumococcal Disease (IPD) during the post-vaccine period (2013–2016).

Age-group	Rate ratio (95% CI)	Cumulative cases prevented (95% CI)	Direct savings (USD, 95% CI)	Indirect savings (USD, 95% CI)
0-4y	0.27 (0.05–3.00)	14 (-2 to 67)	227,087 (71,363 to 618,919)	16,882 (6,893 to 38,718)
5-64y	0.44 (0.31–0.68)	29 (1 to 65)	321,424 (-455,573 to 1,649,171)	12,983 (-3,606 to 33,498)
65y+	0.94 (0.62–1.53)	10 (-16 to 45)	73,395 (-256,856 to 516,864)	4,340 (-10,903 to 23,543)

The rate ratio is presented along with 95% credible intervals (95% CI) for the three age-groups. The predicted cumulative number of prevented cases as of 1 December 2016 is also presented. A negative number indicates that there is a non-zero probability that the vaccine caused more IPD hospitalizations to occur. Direct and indirect savings are presented in constant 2015 USD.

Sensitivity analyses using restricted pre-vaccine periods were not possible as the models did not fit with such a small amount of data. The results did not change with the inclusion of different controls (S15 Fig). Using vaccine-type IPD as the outcome measure resulted in larger estimates of vaccine impact, which ranged from 100% in children zero to four years of age to 68% in adults 65 years of age and older (S16 Fig).

### Cost-effectiveness of PHiD-CV10

The Primary Care Registry was complete until December 31, 2015. Therefore, cost-effectiveness was calculated until that date.

The total cost of introducing PHiD-CV10 into the Icelandic paediatric immunisation programme from 1 January 2011 to 31 December 2015 was 3,451,805 in constant 2015 USD. When direct cost-savings due to reductions in primary care visits for AOM, hospital admissions for pneumonia and hospital admissions for IPD were included, the overall cost of the PHiD-CV10 introduction was -7,463,176 USD (95% CI -16,159,551 USD to -582,135 USD) which is equivalent to a return on investment of 2.1 USD (95% CI 0.2–4.7) for every 1 USD invested. Including the indirect costs, the overall savings PHiD-CV10 introduction was 8,164,894 USD (95% CI 1,004,553 USD to 17,197,959 USD) or 2.4 USD (95% CI 0.3–5.0) for every 1 USD invested into the immunisation program.

Given the observed distribution of costs associated with each AOM visit, the direct savings resulting from vaccine-prevented AOM visits was 1,389,900 USD (95% CI 704,319 USD to 2,201,925 USD). The vaccine introduction prevented 10,911 days of work lost due to AOM (95% CI 5,116 to 18,801), which translates to 194,152 USD (95% CI 78,200 USD to 364,155 USD) in productivity gains (Table 3). Including only direct costs from averted visits and hospitalisations associated with AOM, the ICER was -543 USD (95% CI -1,508 USD to -48 USD) per prevented visit. The corresponding ICER from the societal perspective was -594 USD (95% CI -1,597 USD to -76 USD) per prevented AOM case.

The direct savings from vaccine-prevented pneumonia hospitalisations was 13,330,902 USD (95% CI 933,955 USD to 26,270,332 USD). Assuming no other benefits than preventing pneumonia hospitalisations, and considering only direct costs, the ICER was -5,315 USD (95% CI -8,877 USD to 711 USD) per prevented pneumonia hospitalisation. When the cost-savings due to reductions in AOM visits and hospital admissions for IPD were also included, the ICER per prevented pneumonia hospitalisation was -5,640 USD (95% CI -10,336 USD to -1,032 USD) as of 31 December 2015 (Table 4). Including loss of work resulted in an ICER of -7,440 USD (95% CI -13,701 USD to -1,175 USD) per prevented pneumonia hospitalisation.

The direct savings resulting from vaccine-prevented hospitalisations of IPD was 673,008 USD (95% CI -189,654 USD to 2,081,594 USD). The vaccine programme prevented 1,280 days of work lost (444 to 2,410) due to IPD, which translates to 35,280 USD (95% CI 9,437 USD to 70,609 USD) in productivity gains. When cost-savings due to reductions in AOM visits and hospital admissions for pneumonia were also included, the ICER was -119,992 USD (95% CI -387,183 USD to -9,542 USD) per prevented IPD hospitalization (Table 5). When days of work lost were also considered, the ICER was -130,791 USD (95% CI -416,004 USD to -15,860 USD) per prevented IPD hospitalization.

## Discussion

This population-based time series analysis demonstrated a direct and indirect impact of PHiD-CV10 introduction on AOM, pneumonia and IPD in Iceland. After considering savings due to prevented episodes of pneumococcal infections, the PHiD-CV10 programme was shown to be cost-saving from both the healthcare and societal perspectives. The results were robust to sensitivity analyses and falsification tests, which did not reveal evidence of confounding.

The study is strengthened by its long observation period and the completeness of the underlying data. Six years of pre-vaccine data were used to estimate secular trends occurring before the implementation of the vaccine. Between five and seven years of post-implementation data were included depending on outcome. Both periods are longer than most previous observational studies of PCV impact [6, 7]. The Primary Care Registry of the Directorate of Health contains data on all primary care contacts in the country, and though Landspitali University Hospital is a single center, it is by far the largest acute care hospital and the sole tertiary hospital in Iceland, and includes the Children’s Hospital–Iceland’s only paediatric hospital. In 2017, the total number of non-psychiatric curative care hospital beds in Iceland was 732 (www.statice.is). Of those, 669 (91%) were at Landspitali University Hospital. It provides primary and secondary care for the capital area, approximately 65% of the Icelandic population.

### Impact on acute otitis media

Our study builds upon the literature by providing population-based estimates of direct and indirect PCV impact on AOM that is adjusted for several controls and for secular trends. Furthermore, extensive sensitivity analyses demonstrated that the result was robust to different case definitions of AOM, and a falsification test showed that no spurious decline in urinary tract infections. Two systematic reviews of the impact of PCV on AOM have been published [22, 23]. Of the nine observational studies identified by the reviews, only three adjusted for secular trends [24–26]. Lau et al. used an interrupted time series approach to estimate the sequential impact of PCV7 and PCV13 on otitis media in general practice and reported a 21.8% reduction in the rate of otitis media visits in children younger than 10 years of age. Marom et al. and Grijalva et al. calculated rate ratios of otitis media visits between children younger than two years of age and children three to six years of age and estimated the impact to be 20% and 27% respectively. Only two previous studies have suggested indirect protection of PCV against AOM [8, 9]. Both reported fewer episodes among children younger than four months of age, who were too young to have received direct protection from PCV. Ben-Shimol et al. described a decrease in positive pneumococcal cultures from samples taken from the middle ear of children in this age-group, and Sigurdsson et al. noted fewer primary care diagnosed AOM episodes.

### Impact on pneumonia

Our results were consistent with a large direct and indirect impact on pneumonia hospitalisations. Sensitivity analyses revealed the result to be robust to different case definitions, controls and pre-vaccine periods. A recent systematic review and meta-analysis found that the average direct vaccine impact on clinical pneumonia was 17% (95%CI 11% to 22%) [27]. Of the observational studies identified by the systematic review, three were population-based [28–30]. The methods used in our study are based on Bruhn et al. who demonstrated their method using data on pneumonia hospitalisations before and after the introduction of PCV7 and PHiD-CV10 in five countries; Brazil, Chile, Ecuador, Mexico and the United States [6]. They showed a 14% to 45% impact on pneumonia hospitalisations among children younger than 12 months of age.

Few previous publications have examined the indirect impact of pneumococcal conjugate vaccines on pneumonia hospitalisations among the unvaccinated population. Impact estimates ranged from 3% to 24% among children five to 17 years of age; 0% to 26% among adults 18–39 years of age; and 0% to 19% among adults 40–64 years of age [6, 31–36]. All but two of the studies suggested an impact among adults 65 years of age and older, with estimates ranging from 3% to 15%, though none reached statistical significance.

### Impact on invasive pneumococcal disease

Our findings are largely congruent with previous studies examining the herd-effect of PCV on all-cause IPD. We show a robust indirect protection among individuals five to 64 years of age, after adjusting for any secular trends in the pre-vaccine period, and the result is consistent with visual examination of the raw data (Fig 6). Though our findings are consistent with a slight decrease in IPD hospitalizations among adults 65 and older, the effect is not as obvious. While surprising, this result is consistent with the body of literature which seems to suggest a large and robust impact on vaccine-type IPD in this age-group, but a marginal impact on all-cause IPD [5, 37]. When vaccine-type IPD was used as an outcome measure, the vaccine impact was considerably larger and was robust in all age-groups.

Two systematic reviews identified 262 observational studies that examined the direct and indirect impact of PCV on vaccine-type and all-cause IPD [5, 37]. The publication by Shiri et al. was also a meta-analysis, which used a Bayesian mixed-effects model to translate the included studies into a single estimate. The study demonstrated a yearly post-vaccine risk ratio of vaccine-type IPD of 0.79 (95% CI 0.75–0.81), translating to a mean period to attain a 50% population reduction of vaccine-type IPD of 2.3 years (95% CI 1.9–2.7), and 8.9 years (95% CI 7.8–10.3) to attain a 90% reduction [5].

### Cost-effectiveness of PHiD-CV10

A large number of cost-effectiveness analyses of pneumococcal conjugate vaccines have been published [13, 38, 39]. Our results are quantitatively similar to the body of cost-effectiveness literature of PCV. Most studies show that introducing PCV into national immunization programs is cost-effective when compared to no vaccination. However, our study adds important aspects to prior studies.

We included more granular data than have previously been used in cost-effectiveness analyses of PCV. Because they are in essence predictive models, cost-effectiveness analyses are particularly sensitive to the accuracy of the modelling assumptions [40]. Most prior studies did not collect detailed data on vaccine uptake, serotype coverage, incidence of disease in the population, disease sequelae, or direct and indirect costs [13, 39]. Efficacies were based on the results of randomised controlled trials, but the existence and magnitude of herd-effect and serotype-replacement were usually based on assumptions and expert opinion [13, 39]. Contrast this with our study, in which all inputs were directly measured in the population.

Our study is inherently different from most previous studies, in that it examines the cost-effectiveness of an intervention that has already been introduced. The most obvious strength of a post-implementation ecological design, is that it does not rely on untestable assumptions regarding herd-effect and serotype-replacement, but rather bases them on direct observation. To our knowledge, only two previous studies have reported the post-implementation cost-effectiveness of PCV [15, 16]. Neither study directly estimated the impact of PCV on AOM, pneumonia or IPD but instead relied on previously published estimates or efficacy data.

Our study does have several limits. The results are highly dependent on indirect protection against pneumonia hospitalisations being conferred to older adults, which was supported by the stacked Bayesian time series model and sensitivity analyses, and congruent with the results of several previous studies [6, 31–36]. It should however be noted that the synthetic control model, which was the most conservative of the four included time series models, predicted none to little indirect protection against pneumonia in these age-groups (S8 and S11 Figs), a result consistent with a previous publication using this method [6]. The post-implementation observational nature of the study placed several constraints on the analysis. We did not include sequalae of infections in our analysis as we did not have access to mortality data and sequalae of AOM, pneumonia or IPD are not routinely measured in population registries. Productivity loss was based entirely on assumptions as no Icelandic registry keeps track of days of work lost due to illness. Finally, the time horizon was only five years and it is likely that cost-savings would continue to accrue if the time horizon were increased.

## Conclusions

In this time series analysis of population-based data, we demonstrated a substantial direct impact on AOM in vaccinated children and provided the first published evidence of herd protection against AOM among older unvaccinated children. We showed a large decrease in pneumonia hospitalisations among both vaccinated and unvaccinated members of the population, children and adults, thus confirmed previous papers showing an indirect impact. Our results demonstrate that initially expensive vaccine interventions can be shown to produce such a decrease in health care consumption that the resulting cost-savings offset the initial expenditure–and simultaneously reduced suffering in the population. Our study highlights the importance of careful post-implementation studies. These function both as a tool to validate and calibrate the predictions made by pre-implementation cost-effectiveness studies that rely heavily on unverifiable assumptions, and provide evidence of vaccine benefit for policy makers.

## Supporting information

S1 Appendix (DOCX)

S1 Fig The number of primary care visits among children younger than 20 years of age per calendar-month from 1 January 2005 to 31 December 2015. Children are divided into seven age-groups, listed in the figure legend. Panel A shows the number of monthly visits due to acute otitis media and its complications (AOM). Panels B and C, depict the standardized number of monthly AOM visits (Panel B) and all other visits (Panel C) per age-group. The Y-axis represents the number of standard deviations the observed visits are from the mean of the entire period, for each diagnosis and age-group. The horisontal dashed lines represent values that are zero standard deviations from the mean, and the vertical dotted lines represent the beginning of the vaccine intervention. Locally estimated scatter-plot smoothing (LOESS) produced an average trend. Panels B and C suggest that the number of both AOM visits and all other visits decreased in the post-vaccine period, and that AOM visits decreased to a larger degree.
(TIF)

S2 Fig The observed and predicted number of visits for Acute Otitis Media and its complications (AOM) from 1 January 2005 to 31 December 2015 for each age-group. Observed visits are illustrated as black points and the predicted number of visits are drawn as lines for each of the component models. The start of the vaccine period is delineated with a vertical black dotted line. Each component model was fitted to the observed visits in the pre-vaccine period, and then used to predict the number of visits in the post-vaccine period, had the vaccine not been introduced. The distance between the observed and predicted visits for each calendar-month is depicted with a thin black line. Longer distances suggest a larger discrepancy. Note that the scale of the Y-axis differ between age-groups.
(TIF)

S3 Fig The estimated rate ratio between the observed and predicted number of Acute Otitis Media (AOM) visits in the post-vaccine period by model and the number of pre-vaccine years. Each age-group is shown separately on the X-axis. An additional pre-vaccine year is added from left to right, starting with the period 2005–2007 and ending with the full pre-vaccine period 2005–2010 that was used in the main analysis. The top frame shows the estimates for the final stacked model. The results are largely invariant to the number of pre-vaccine years, with a slight trend towards decreasing impact as more years are added.
(TIF)

S4 Fig The estimated rate ratio between the observed and predicted number of Acute Otitis Media (AOM) visits in the post-vaccine period for the synthetic control model. The leftmost point and confidence interval represents the full synthetic model used in the analysis. The same colored label shows the top control and its associated inclusion probability in the Bayesian variable selection process. From left to right, the top control is removed, the model is refitted on the remaining controls and the corresponding rate ratio illustrated with a point and interval. The results are largely invariant to the controls used.
(TIF)

S5 Fig The estimated rate ratio between the observed and predicted number of Acute Otitis Media (AOM) visits in the post-vaccine period for the final stacked model, using different case-definitions. The case-definition used in the main analysis is shown with a red point and intervals. The green point represents the same International Classification of Diseases, 10th revision (ICD-10) codes but only those resulting in an antimicrobial prescription. Finally the blue point represents only H66: Suppurative otitis media. The results are largely invariant to the case-definition with the exception of one year old children.
(TIF)

S6 Fig The population impact of the pneumococcal conjugate vaccine (PHiD-CV10) on outpatient visits for Urinary Tract Infections (UTI) is summarized. In Panel A, the estimated 12-month rolling rate ratio between the observed and predicted number of UTI visits in the post-vaccine period (2011–2015) is shown per age-group. Panel B depicts the cumulative number of prevented UTI visits during the post-vaccine period (2011–2015) for each age-group along with 95% credible intervals. The total cumulative prevented UTI visits regardless of age-group is shown in Panel C. As expected, there was no discernible impact.
(TIF)

S7 Fig The monthly number of hospital admissions for pneumonia and hospitalisations regardless of diagnosis from 1 January 2005 to 31 December 2017. Panel A shows the monthly number of pneumonia hospitalisations. Panels B and C depict the standardized monthly number of pneumonia hospitalisations (Panel B) and all other hospitalisations (Panel C) per age-group. The Y-axis shows how many standard deviations from the mean the observed hospitalizations are by diagnosis and age-group. The horisontal dashed lines represents values that are zero standard deviations from the mean and the vertical dotted lines represent the start of the vaccine intervention. Locally estimated scatter-plot smoothing (LOESS) is used to produce an average trend.
(TIF)

S8 Fig The observed and predicted number of pneumonia hospitalizations from 1 January 2005 to 31 December 2017 for each age-group. Observed cases are illustrated as black points and the predicted number of cases are drawn as lines for each of the component models. The start of the vaccine period is delineated with a vertical black dashed line. Each component model was fitted to the observed number of cases in the pre-vaccine period. They were then used to predict the number of cases that would have occurred in the post-vaccine period, had the vaccine not been introduced. The distance between the observed and predicted cases for each calendar-month is depicted with a thin black line. Longer distances suggest a larger discrepancy between observed and predicted cases. Note that the scale of the Y-axis differ between age-groups.
(TIF)

S9 Fig The estimated rate ratio between the observed and predicted number of pneumonia hospitalisations in the post-vaccine period for the final stacked model using different case-definitions. The case-definition used in the main analysis is shown with a red point and intervals. The green point represents International Classification of Diseases, 10th revision (ICD-10) codes more specific to bacterial pneumonia. Finally the blue point represents the more specific ICD-10 definition of bacterial pneumonia, but only includes those hospitalizations in which radiographical and microbiological testing was performed. Using the most specific definition of pneumonia (blue), the impact of the conjugate vaccine (PHiD-CV) is significantly larger in all age-groups.
(TIF)

S10 Fig The estimated rate ratio between the observed and predicted number of pneumonia hospitalisations in the post-vaccine period for the synthetic control model. The leftmost point and confidence interval represents the full synthetic model used in the analysis. The same colored label shows the top control and its associated inclusion probability in the Bayesian variable selection process. From left to right, the top control is removed, the model is refitted on the remaining controls, and the corresponding rate ratio illustrated with a point and interval. The results are largely invariant to the controls used.
(TIF)

S11 Fig The estimated rate ratio between the observed and predicted number of pneumonia hospitalisations in the post-vaccine period by model and the number of pre-vaccine years. Each age-group is shown separately on the X-axis. An additional pre-vaccine year is added from left to right, starting with the period 2005–2007 and ending with the full pre-vaccine period 2005–2010 that was used in the main analysis. The top frame shows the estimates for the final stacked model. The results are largely invariant to the number of pre-vaccine years. However, when only 2005–2007 are included, the estimates are severely unstable in the principal component analysis (PCA) model. The PCA model was given undue weight in the model stacking procedure, resulting in the same instability in the final stacked model. Despite this, the figure does not suggest that the inclusion of 2009 has large effects on the results. The 2009 influenza pandemic therefore, does not seem to unduly influence the results.
(TIF)

S12 Fig The population impact of the pneumococcal conjugate vaccine (PHiD-CV10) on Urinary Tract Infections (UTI) hospitalisations. In Panel A, the estimated 12-month rolling rate ratio between the observed and predicted number of UTI visits in the post-vaccine period (2011–2015) is shown per age-group. Panel B depicts the cumulative number of prevented UTI visits during the post-vaccine period (2011–2015), for each age-group along with 95% credible intervals. The total cumulative prevented UTI visits regardless of age-group is shown in Panel C. As expected, there was no discernible impact.
(TIF)

S13 Fig The number of hospitalizations per year-quarter from 1 January 2005 to 31 December 2016. The population is divided into three age-groups, listed in the figure’s legend. Panel A shows the absolute quarterly number of hospital admissions due to invasive pneumococcal disease (IPD), regardless of serotype. Panels B and C, depict the standardized quarterly number of IPD hospitalizations (Panel B) and all-cause hospitalizations (Panel C) per age-group. The Y-axis represents the number of standard deviations from the mean hospitalizations for each quarter and each age-group. The horizontal dotted lines represent values that are zero standard deviations from the mean and the vertical dotted lines represent the start of the vaccine intervention. Locally estimated scatter-plot smoothing (LOESS) produced an average trend. Panels B and C have been magnified to emphasize the interpretation of the trend line. Panels B and C show that standardized hospitalizations for IPD decreased in all age-groups, relative to the standardized hospitalizations, regardless of cause.
(TIF)

S14 Fig The observed and predicted number of IPD hospitalizations from 1 January 2005 to 31 December 2016, for each age-group. Observed cases are illustrated as black points, and predicted number of cases are drawn as lines for each of the component models. The start of the vaccine period is delineated with a vertical black dotted line. Each component model was fitted to the observed number of cases in the pre-vaccine period. They were then used to predict the number of cases that would have occurred in the post-vaccine period, had the vaccine not been introduced. The distance between the observed and predicted cases for each year-quarter is depicted with a thin black line. Longer distances suggest a larger discrepancy between observed and predicted cases.
(TIF)

S15 Fig The estimated rate ratio between the observed and predicted number of hospitalizations for invasive pneumococcal disease in the post-vaccine period for the synthetic control model. The leftmost point and confidence interval represents the full synthetic model used in the analysis. The same colored label shows the top control and its associated inclusion probability in the Bayesian variable selection process. From left to right, the top control is removed, the model is refitted on the remaining controls and the corresponding rate ratio illustrated with a point and interval. The results are largely invariant to the controls used.
(TIF)

S16 Fig The estimated rate ratio between the observed and predicted number of hospitalizations for Invasive Pneumococcal Disease (IPD) in the post-vaccine period for the final stacked model using different case-definitions. Culture- or PCR-confirmed IPD regardless of serotype, the case-definition used in the main analysis, is shown with a red point and intervals. The green point represents vaccine-type IPD. The number of nonvaccine-type IPD in the pre-vaccine period was not large enough to fit any of the time series models. The figure shows that the impact of the 10-valent pneumococcal Haemophilus influenzae Protein D conjugate vaccine (PHiD-CV) on vaccine-type is considerable in all age-groups.
(TIF)

S1 Table The weights used to produce the final stacked model from the component models. (DOCX)
