# Supplementary material for: The impact and cost-effectiveness of introducing the 10-valent pneumococcal conjugate vaccine into the paediatric immunisation programme in Iceland—A population-based time series analysis
Source: PLoS One. 2021 Apr 8;16(4):e0249497. doi: 10.1371/journal.pone.0249497 (PMC8031404; doi:10.1371/journal.pone.0249497)
Supplement: S1 Appendix — (DOCX) [file pone.0249497.s001.docx]

## S1 Appendix.

## Methods in detail

Four independent models that described the secular trend of pneumococcal infections were fitted. The simplest model was an interrupted time series (ITS) model without an offset term. Calender-month effects were accounted for using dummy variables. The ITS model used the pre-vaccine period to estimate the trend. It predicted the monthly number of cases of the disease category, assuming the pre-vaccine trend would have continued if vaccination had not occurred. A second ITS model was estimated, which included an offset term of all non-respiratory visits. This model used the pre-vaccine period to estimate the relationship between the outcome of interest and all non-respiratory visits. It also predicted the occurrence of disease in the post-vaccine period by incorporating the observed number of non-respiratory visits. The assumption was made that the relationship between the disease category and non-respiratory visits would not have changed had vaccination not occurred. The third model included synthetic controls as covariates, and employed Bayesian variable selection to choose which of them to include (Bruhn et al. [2017](#ref-Bruhn2017)). The prior for each synthetic control was set as a Dirac spike with a point-mass at zero (Dvorzak and Wagner [2019](#ref-R-pogit)). The pre-vaccine period was used to estimate the relationship between the synthetic controls and the outcome of interest, and to select the optimal controls. This relationship predicted the trend in the post-vaccine period, had vaccination not occurred. Finally, a two-step model was fitted, using a seasonal and trend decomposition (STL) and principal component analysis (PCA) (Shioda et al. [2018](#ref-Shioda2018a)). STL was utilized to extract a smoothed trend for each of the synthetic controls. PCA was then applied to extract the first principal component, which was inserted as a covariate in the final prediction model. Using data from the pre-vaccine period, leave-one-out cross-validation (LOOCV) was used to calibrate the models and calculate the average point-wise likelihood for each model, diagnostic category and age-group. The average point-wise likelihoods were the weights in a Bayesian model-stacking procedure to produce the final stacked model for the analysis.
