# Supplementary figures and images for: The impact and cost-effectiveness of introducing the 10-valent pneumococcal conjugate vaccine into the paediatric immunisation programme in Iceland—A population-based time series analysis
Source: PLoS One. 2021 Apr 8;16(4):e0249497. doi: 10.1371/journal.pone.0249497 (PMC8031404; doi:10.1371/journal.pone.0249497)

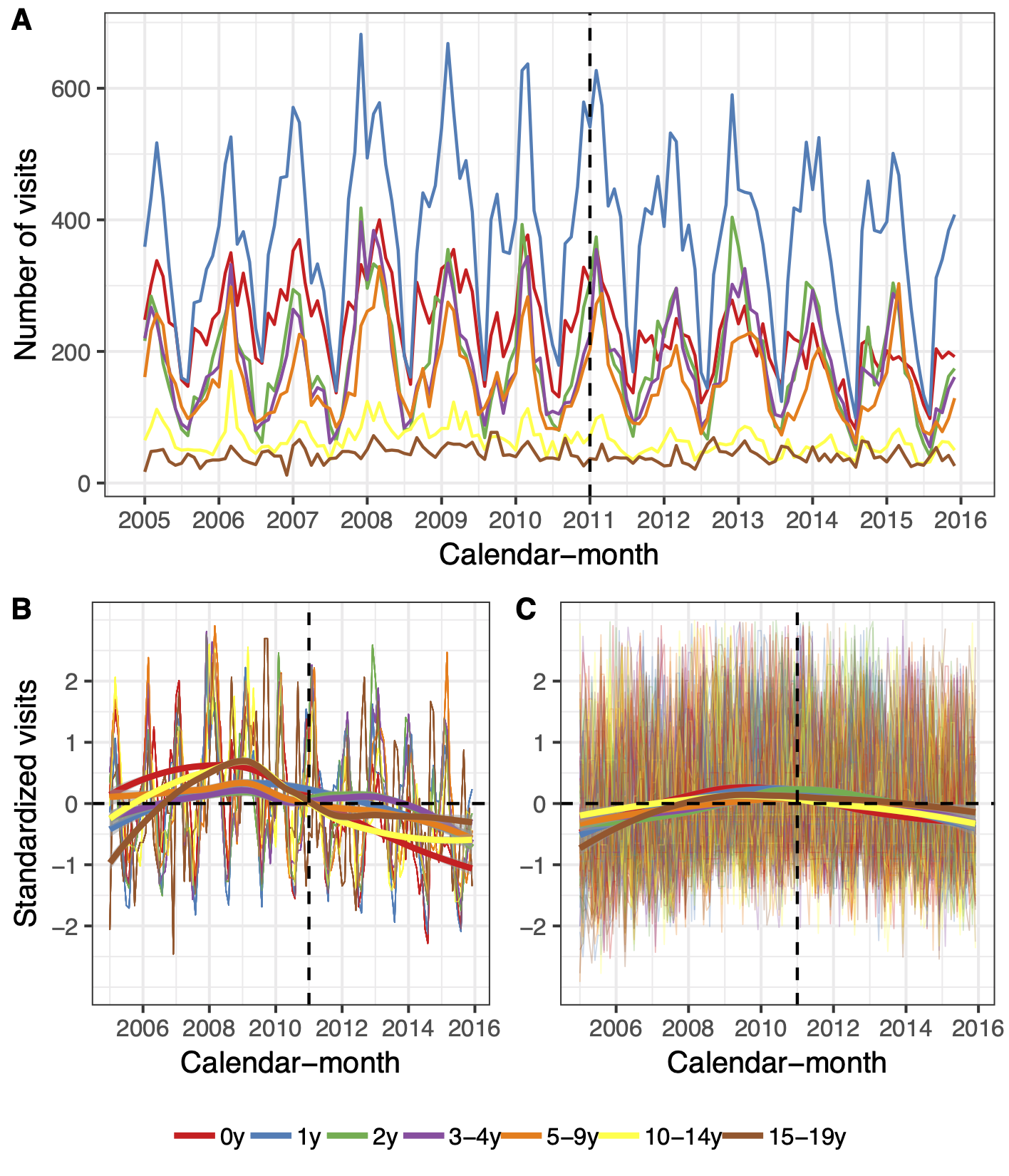

Supplement: S1 Fig — Children are divided into seven age-groups, listed in the figure legend. Panel A shows the number of monthly visits due to acute otitis media and its complications (AOM). Panels B and C, depict the standardized number of monthly AOM visits (Panel B) and all other visits (Panel C) per age-group. The Y-axis represents the number of standard deviations the observed visits are from the mean of the entire period, for each diagnosis and age-group. The horisontal dashed lines represent values that are zero standard deviations from the mean, and the vertical dotted lines represent the beginning of the vaccine intervention. Locally estimated scatter-plot smoothing (LOESS) produced an average trend. Panels B and C suggest that the number of both AOM visits and all other visits decreased in the post-vaccine period, and that AOM visits decreased to a larger degree. (TIF) [file pone.0249497.s002.tif]

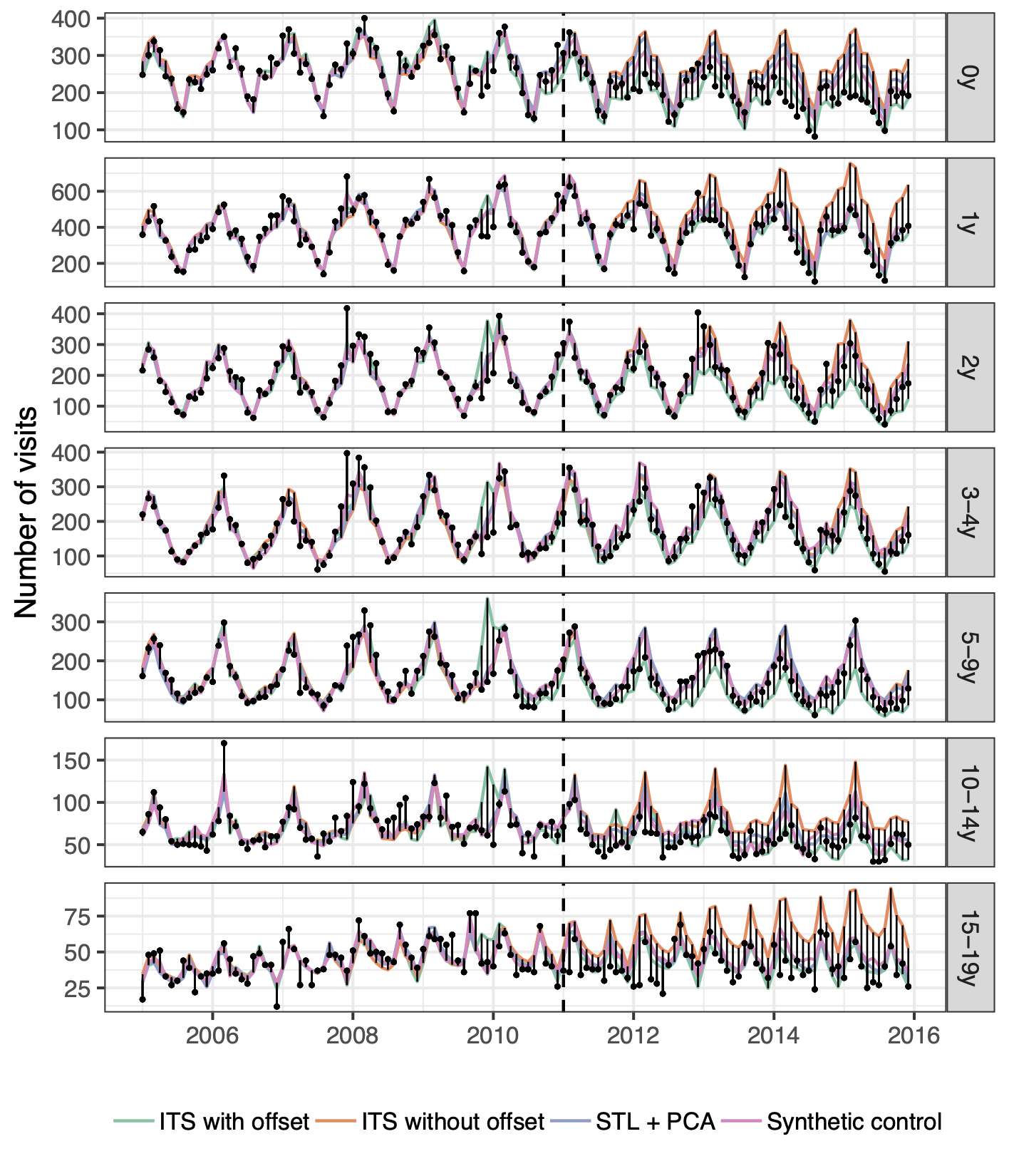

Supplement: S2 Fig — The observed and predicted number of visits for Acute Otitis Media and its complications (AOM) from 1 January 2005 to 31 December 2015 for each age-group. Observed visits are illustrated as black points and the predicted number of visits are drawn as lines for each of the component models. The start of the vaccine period is delineated with a vertical black dotted line. Each component model was fitted to the observed visits in the pre-vaccine period, and then used to predict the number of visits in the post-vaccine period, had the vaccine not been introduced. The distance between the observed and predicted visits for each calendar-month is depicted with a thin black line. Longer distances suggest a larger discrepancy. Note that the scale of the Y-axis differ between age-groups. (TIF) [file pone.0249497.s003.tif]

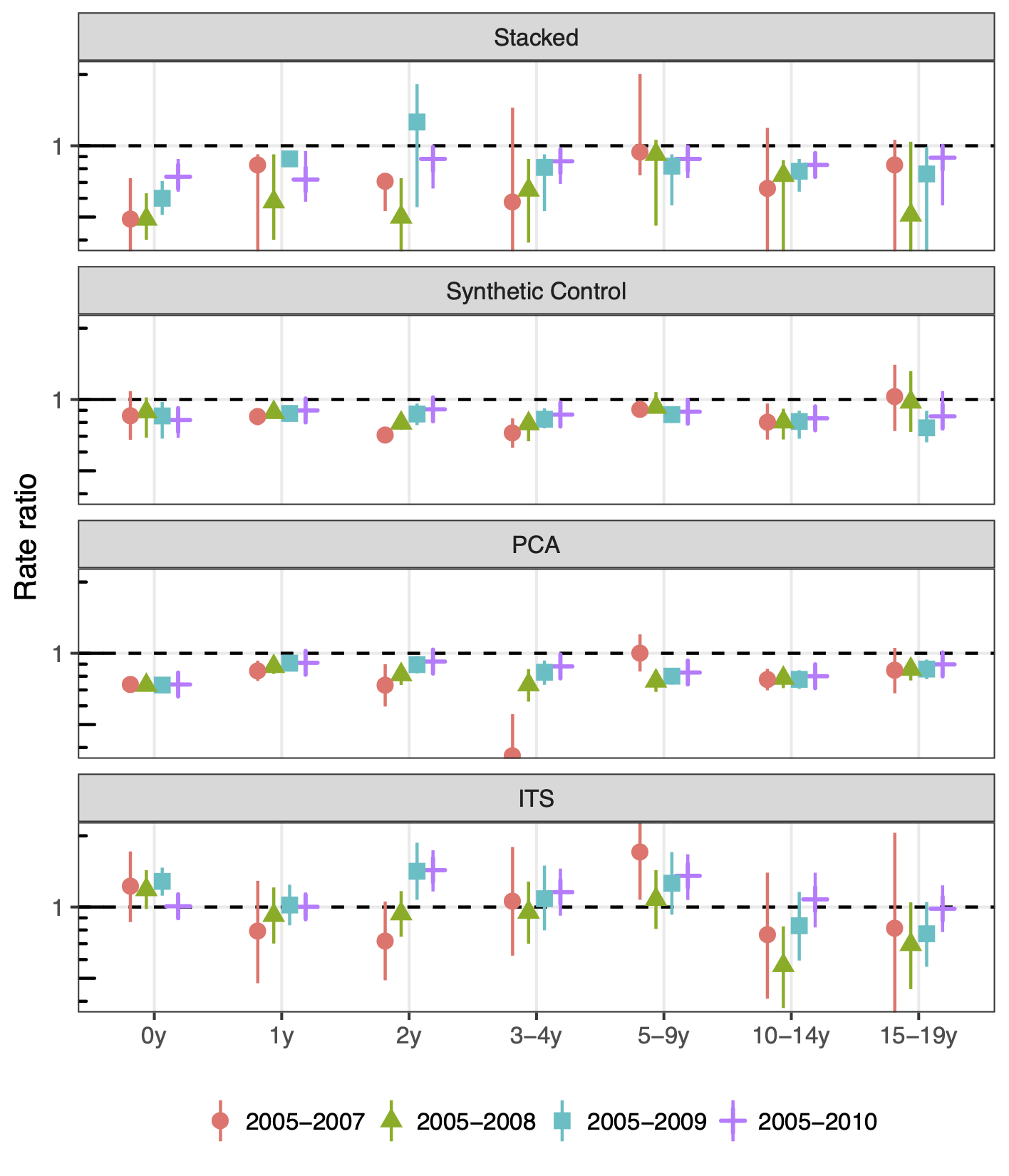

Supplement: S3 Fig — The estimated rate ratio between the observed and predicted number of Acute Otitis Media (AOM) visits in the post-vaccine period by model and the number of pre-vaccine years. Each age-group is shown separately on the X-axis. An additional pre-vaccine year is added from left to right, starting with the period 2005–2007 and ending with the full pre-vaccine period 2005–2010 that was used in the main analysis. The top frame shows the estimates for the final stacked model. The results are largely invariant to the number of pre-vaccine years, with a slight trend towards decreasing impact as more years are added. (TIF) [file pone.0249497.s004.tif]

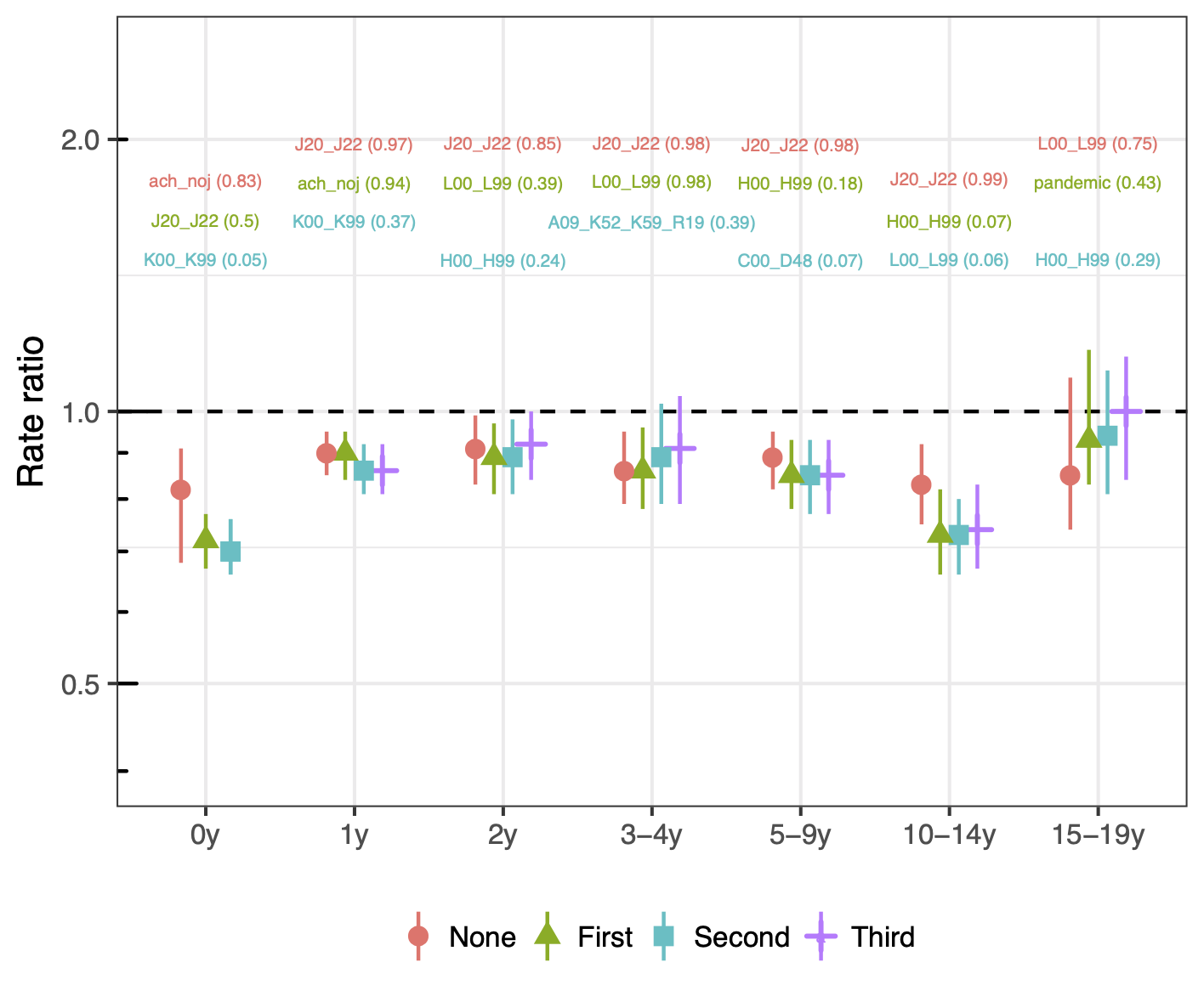

Supplement: S4 Fig — The leftmost point and confidence interval represents the full synthetic model used in the analysis. The same colored label shows the top control and its associated inclusion probability in the Bayesian variable selection process. From left to right, the top control is removed, the model is refitted on the remaining controls and the corresponding rate ratio illustrated with a point and interval. The results are largely invariant to the controls used. (TIF) [file pone.0249497.s005.tif]

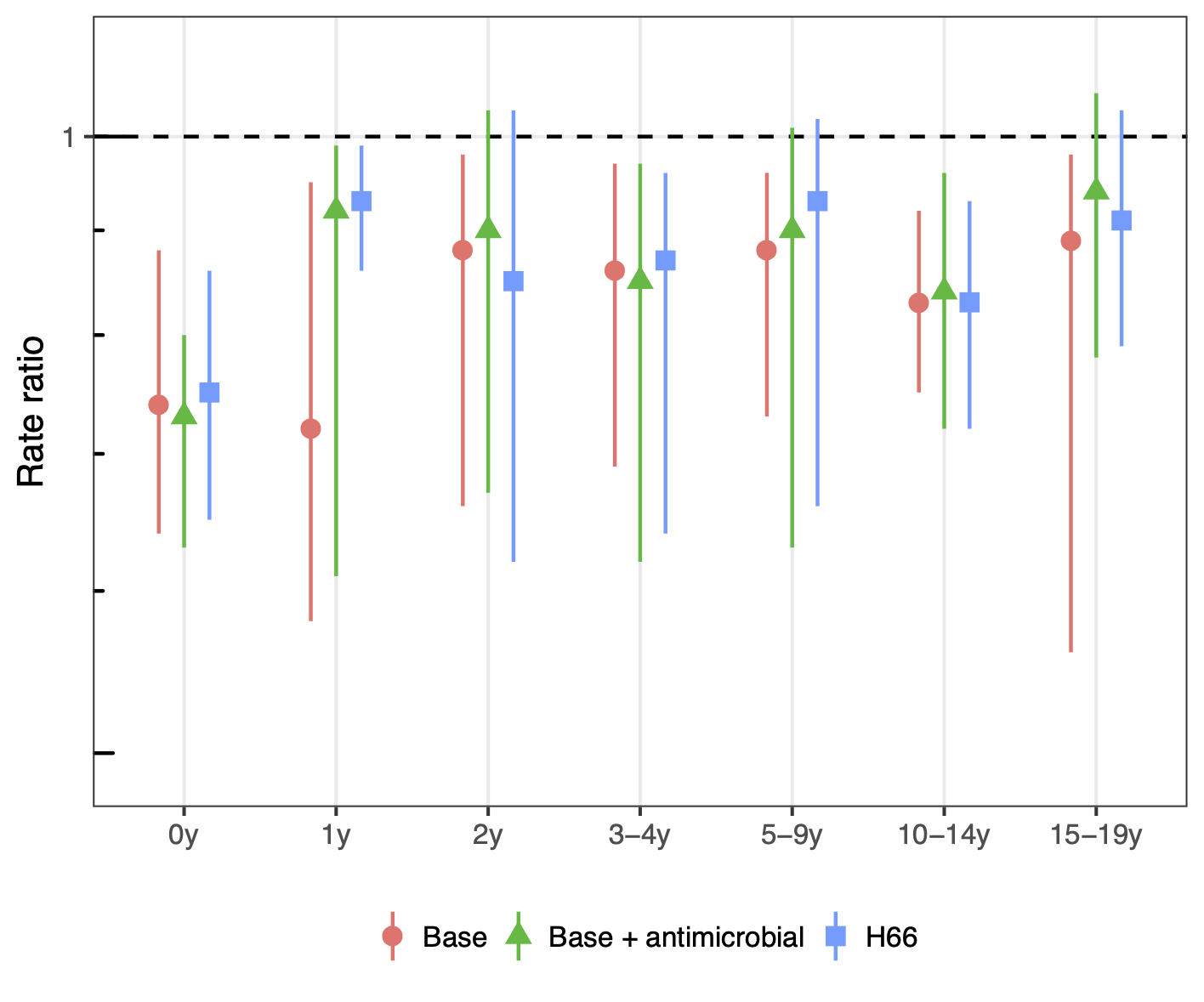

Supplement: S5 Fig — The estimated rate ratio between the observed and predicted number of Acute Otitis Media (AOM) visits in the post-vaccine period for the final stacked model, using different case-definitions. The case-definition used in the main analysis is shown with a red point and intervals. The green point represents the same International Classification of Diseases, 10th revision (ICD-10) codes but only those resulting in an antimicrobial prescription. Finally the blue point represents only H66: Suppurative otitis media. The results are largely invariant to the case-definition with the exception of one year old children. (TIF) [file pone.0249497.s006.tif]

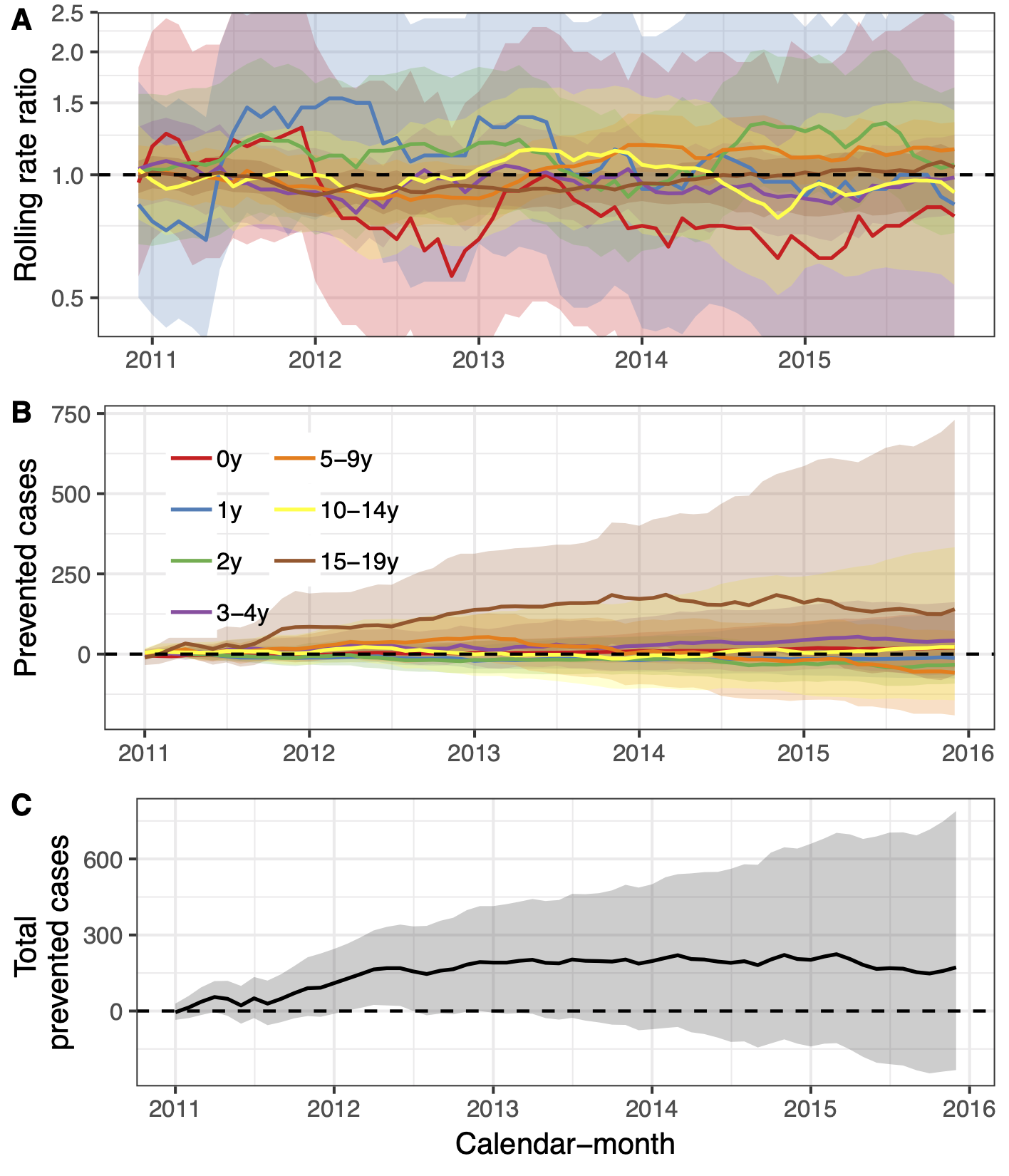

Supplement: S6 Fig — In Panel A, the estimated 12-month rolling rate ratio between the observed and predicted number of UTI visits in the post-vaccine period (2011–2015) is shown per age-group. Panel B depicts the cumulative number of prevented UTI visits during the post-vaccine period (2011–2015) for each age-group along with 95% credible intervals. The total cumulative prevented UTI visits regardless of age-group is shown in Panel C. As expected, there was no discernible impact. (TIF) [file pone.0249497.s007.tif]

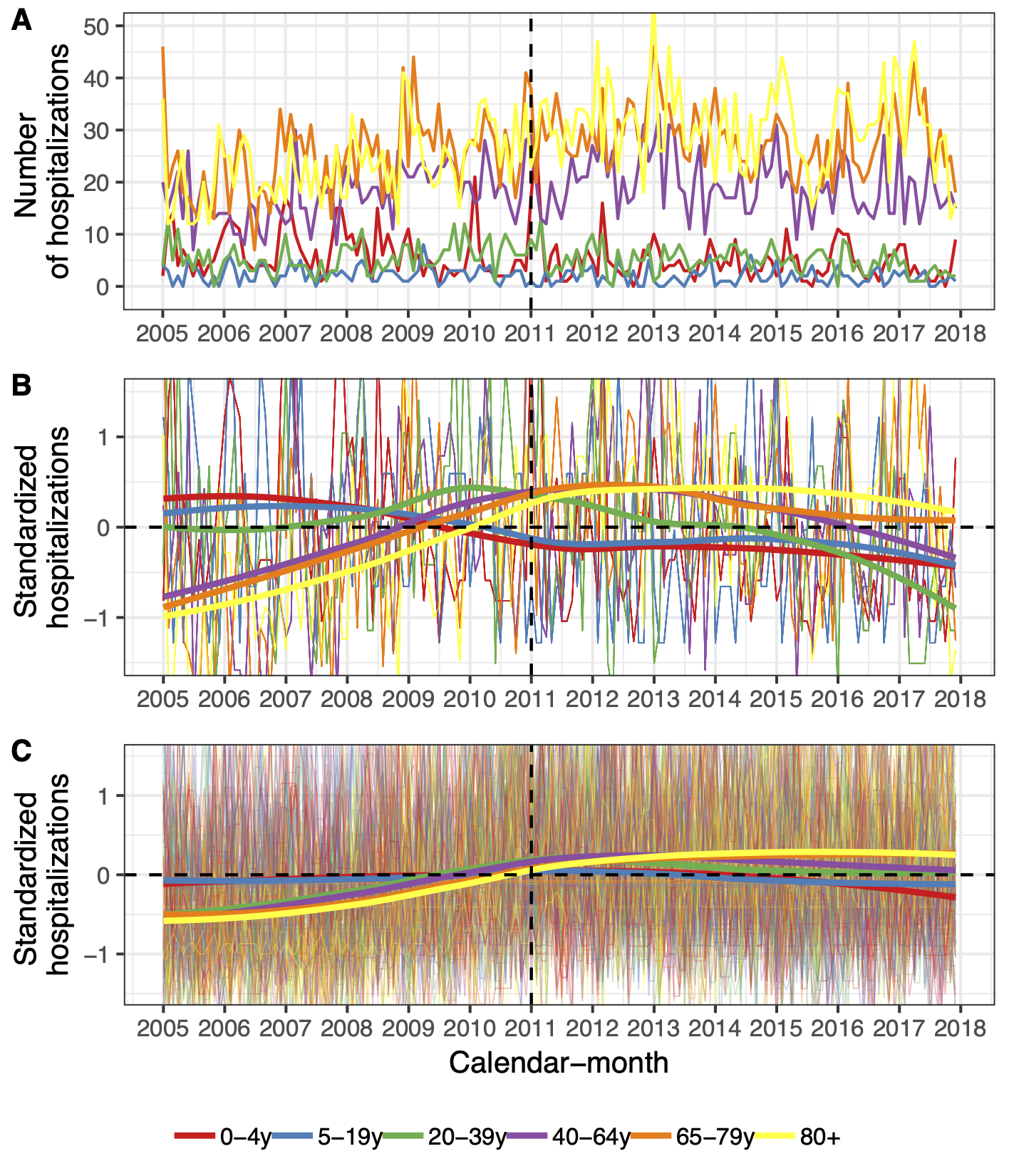

Supplement: S7 Fig — The monthly number of hospital admissions for pneumonia and hospitalisations regardless of diagnosis from 1 January 2005 to 31 December 2017. Panel A shows the monthly number of pneumonia hospitalisations. Panels B and C depict the standardized monthly number of pneumonia hospitalisations (Panel B) and all other hospitalisations (Panel C) per age-group. The Y-axis shows how many standard deviations from the mean the observed hospitalizations are by diagnosis and age-group. The horisontal dashed lines represents values that are zero standard deviations from the mean and the vertical dotted lines represent the start of the vaccine intervention. Locally estimated scatter-plot smoothing (LOESS) is used to produce an average trend. (TIF) [file pone.0249497.s008.tif]

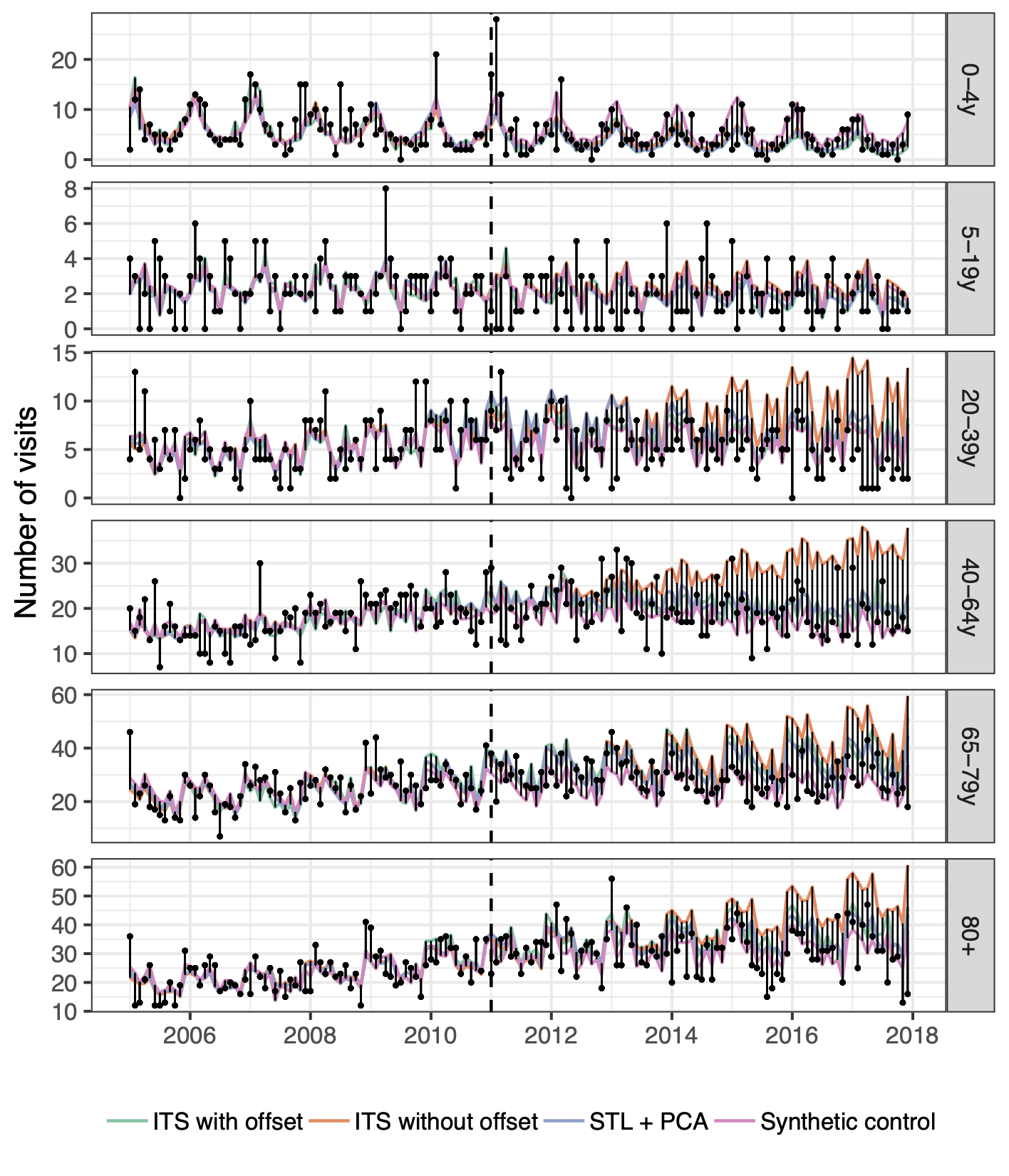

Supplement: S8 Fig — The observed and predicted number of pneumonia hospitalizations from 1 January 2005 to 31 December 2017 for each age-group. Observed cases are illustrated as black points and the predicted number of cases are drawn as lines for each of the component models. The start of the vaccine period is delineated with a vertical black dashed line. Each component model was fitted to the observed number of cases in the pre-vaccine period. They were then used to predict the number of cases that would have occurred in the post-vaccine period, had the vaccine not been introduced. The distance between the observed and predicted cases for each calendar-month is depicted with a thin black line. Longer distances suggest a larger discrepancy between observed and predicted cases. Note that the scale of the Y-axis differ between age-groups. (TIF) [file pone.0249497.s009.tif]

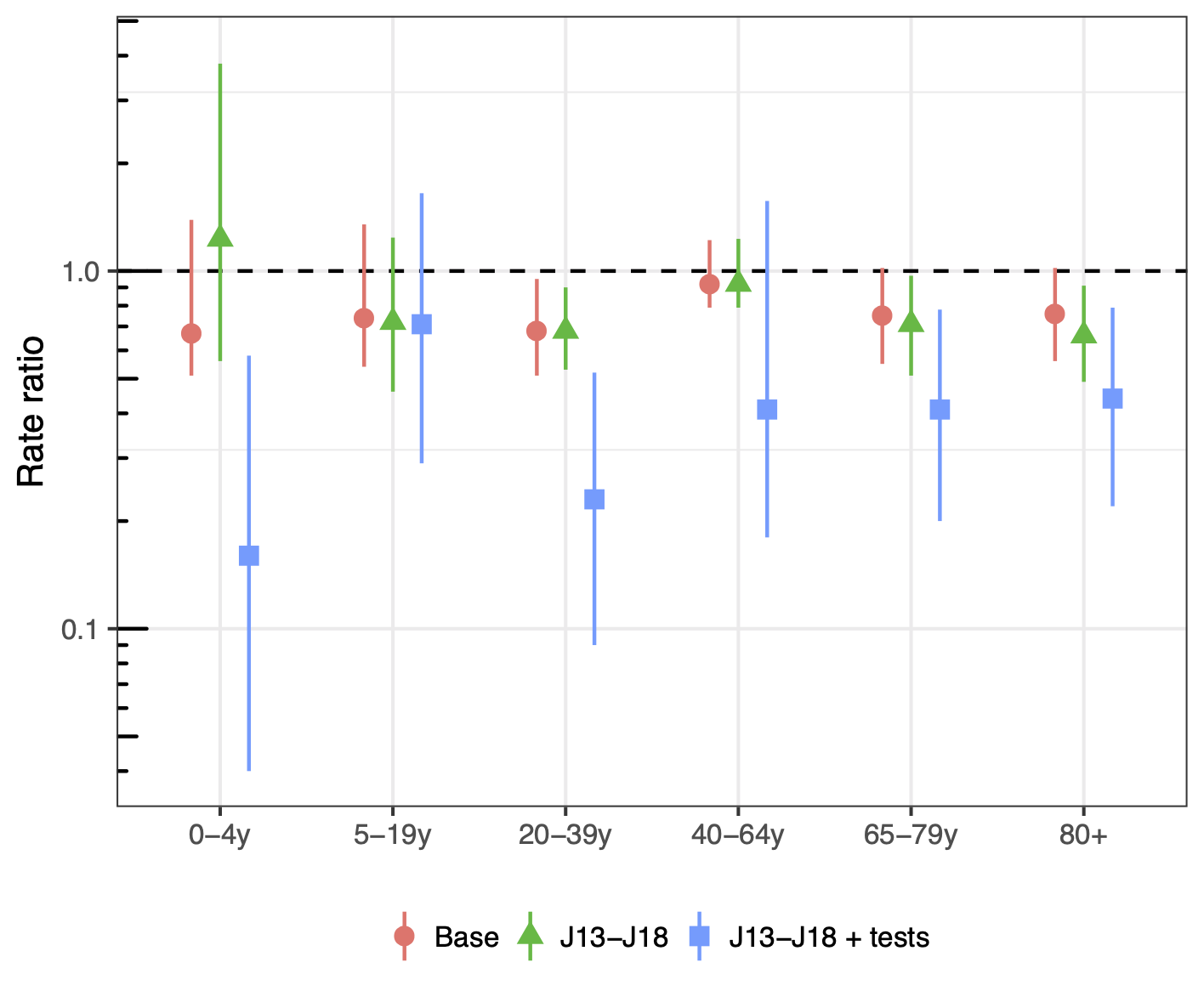

Supplement: S9 Fig — The case-definition used in the main analysis is shown with a red point and intervals. The green point represents International Classification of Diseases, 10th revision (ICD-10) codes more specific to bacterial pneumonia. Finally the blue point represents the more specific ICD-10 definition of bacterial pneumonia, but only includes those hospitalizations in which radiographical and microbiological testing was performed. Using the most specific definition of pneumonia (blue), the impact of the conjugate vaccine (PHiD-CV) is significantly larger in all age-groups. (TIF) [file pone.0249497.s010.tif]

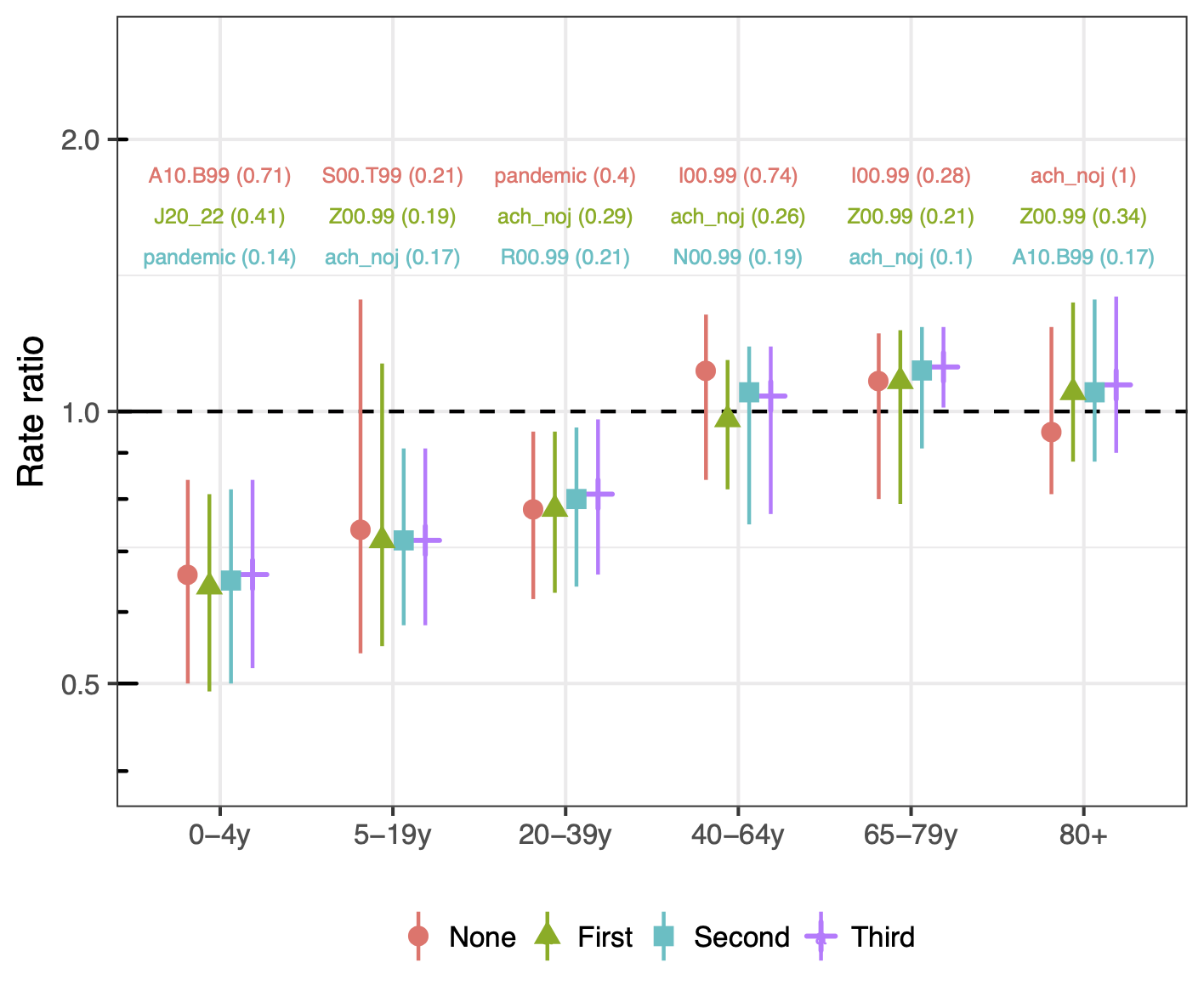

Supplement: S10 Fig — The estimated rate ratio between the observed and predicted number of pneumonia hospitalisations in the post-vaccine period for the synthetic control model. The leftmost point and confidence interval represents the full synthetic model used in the analysis. The same colored label shows the top control and its associated inclusion probability in the Bayesian variable selection process. From left to right, the top control is removed, the model is refitted on the remaining controls, and the corresponding rate ratio illustrated with a point and interval. The results are largely invariant to the controls used. (TIF) [file pone.0249497.s011.tif]

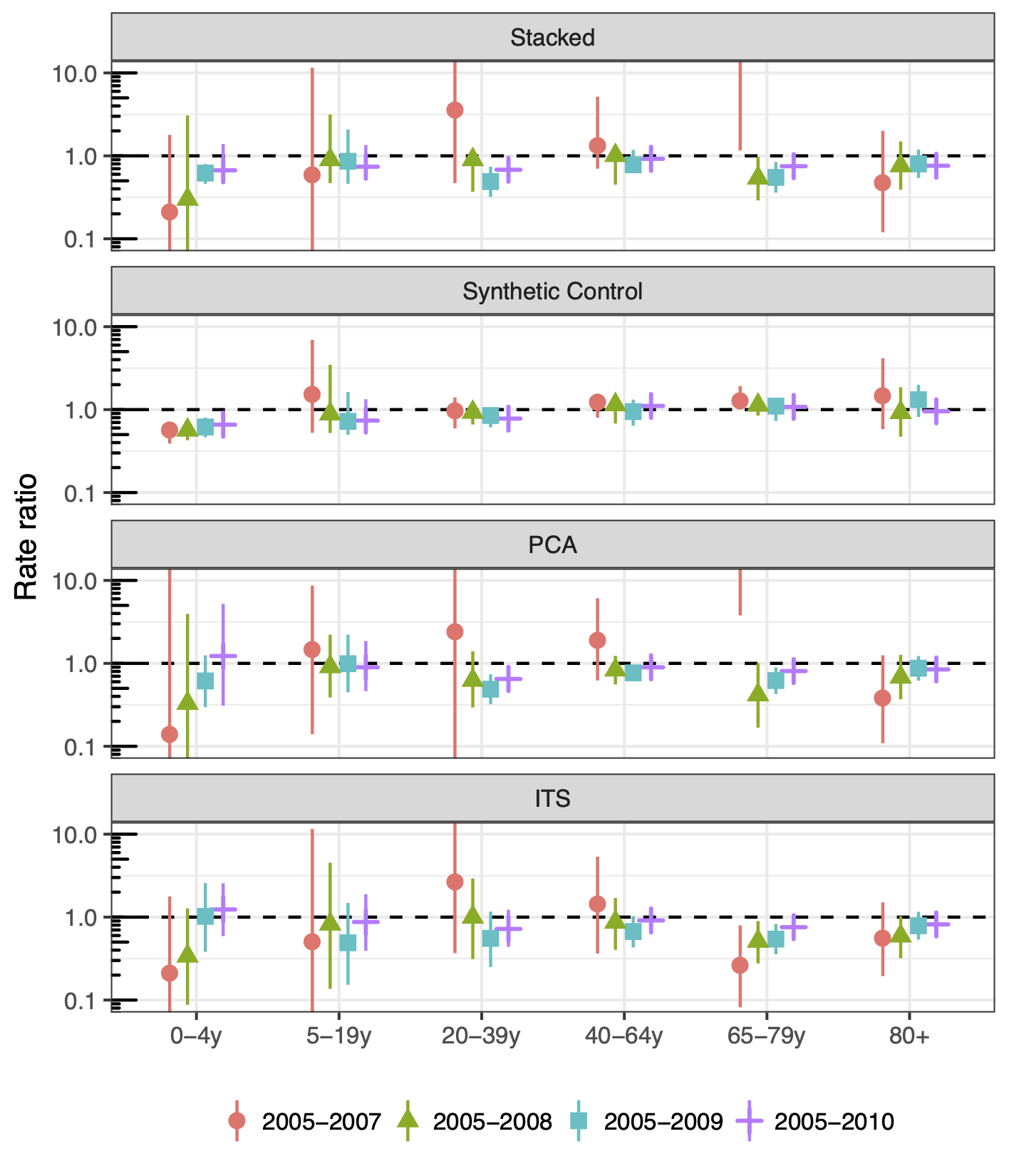

Supplement: S11 Fig — Each age-group is shown separately on the X-axis. An additional pre-vaccine year is added from left to right, starting with the period 2005–2007 and ending with the full pre-vaccine period 2005–2010 that was used in the main analysis. The top frame shows the estimates for the final stacked model. The results are largely invariant to the number of pre-vaccine years. However, when only 2005–2007 are included, the estimates are severely unstable in the principal component analysis (PCA) model. The PCA model was given undue weight in the model stacking procedure, resulting in the same instability in the final stacked model. Despite this, the figure does not suggest that the inclusion of 2009 has large effects on the results. The 2009 influenza pandemic therefore, does not seem to unduly influence the results. (TIF) [file pone.0249497.s012.tif]

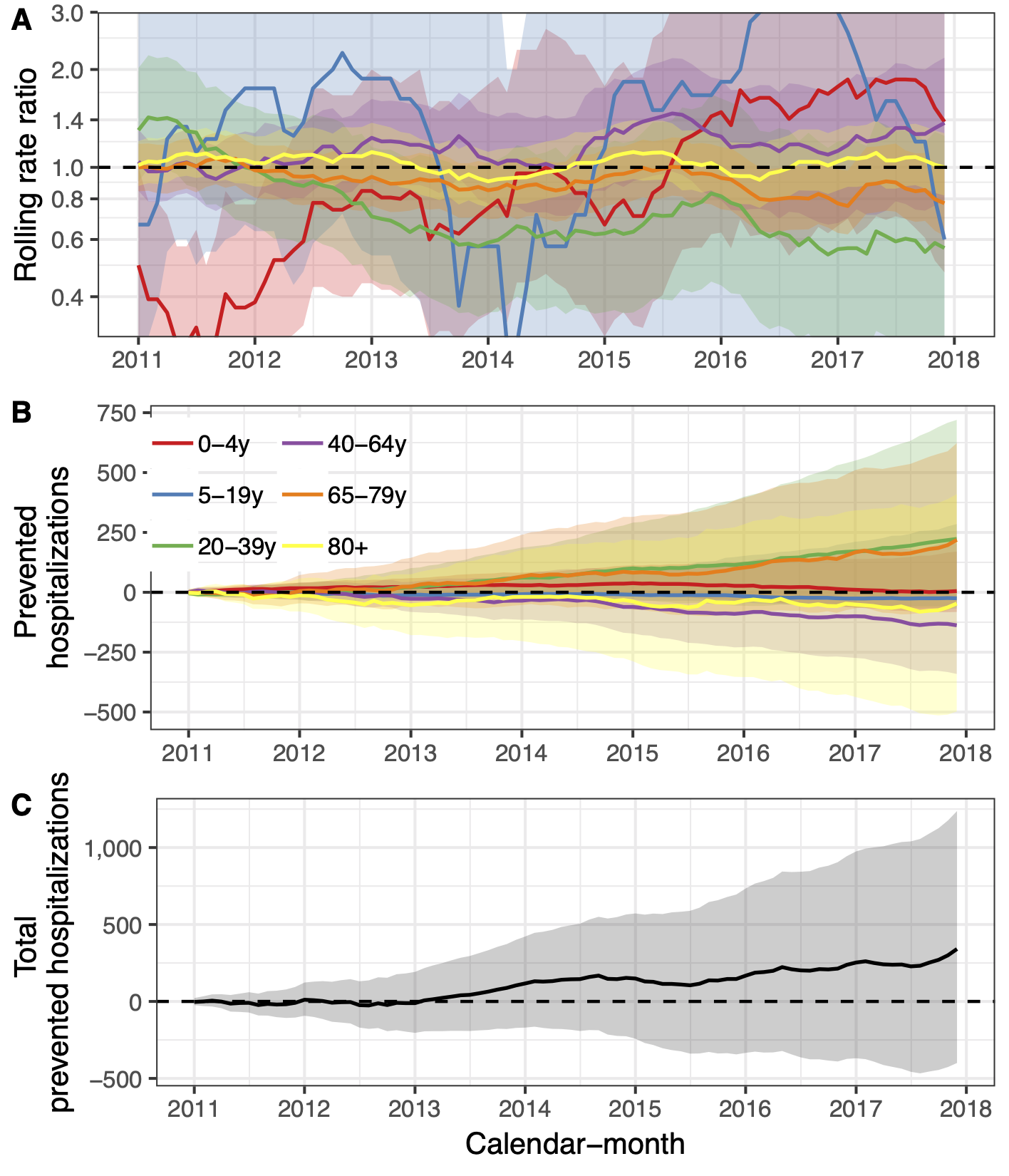

Supplement: S12 Fig — The population impact of the pneumococcal conjugate vaccine (PHiD-CV10) on Urinary Tract Infections (UTI) hospitalisations. In Panel A, the estimated 12-month rolling rate ratio between the observed and predicted number of UTI visits in the post-vaccine period (2011–2015) is shown per age-group. Panel B depicts the cumulative number of prevented UTI visits during the post-vaccine period (2011–2015), for each age-group along with 95% credible intervals. The total cumulative prevented UTI visits regardless of age-group is shown in Panel C. As expected, there was no discernible impact. (TIF) [file pone.0249497.s013.tif]

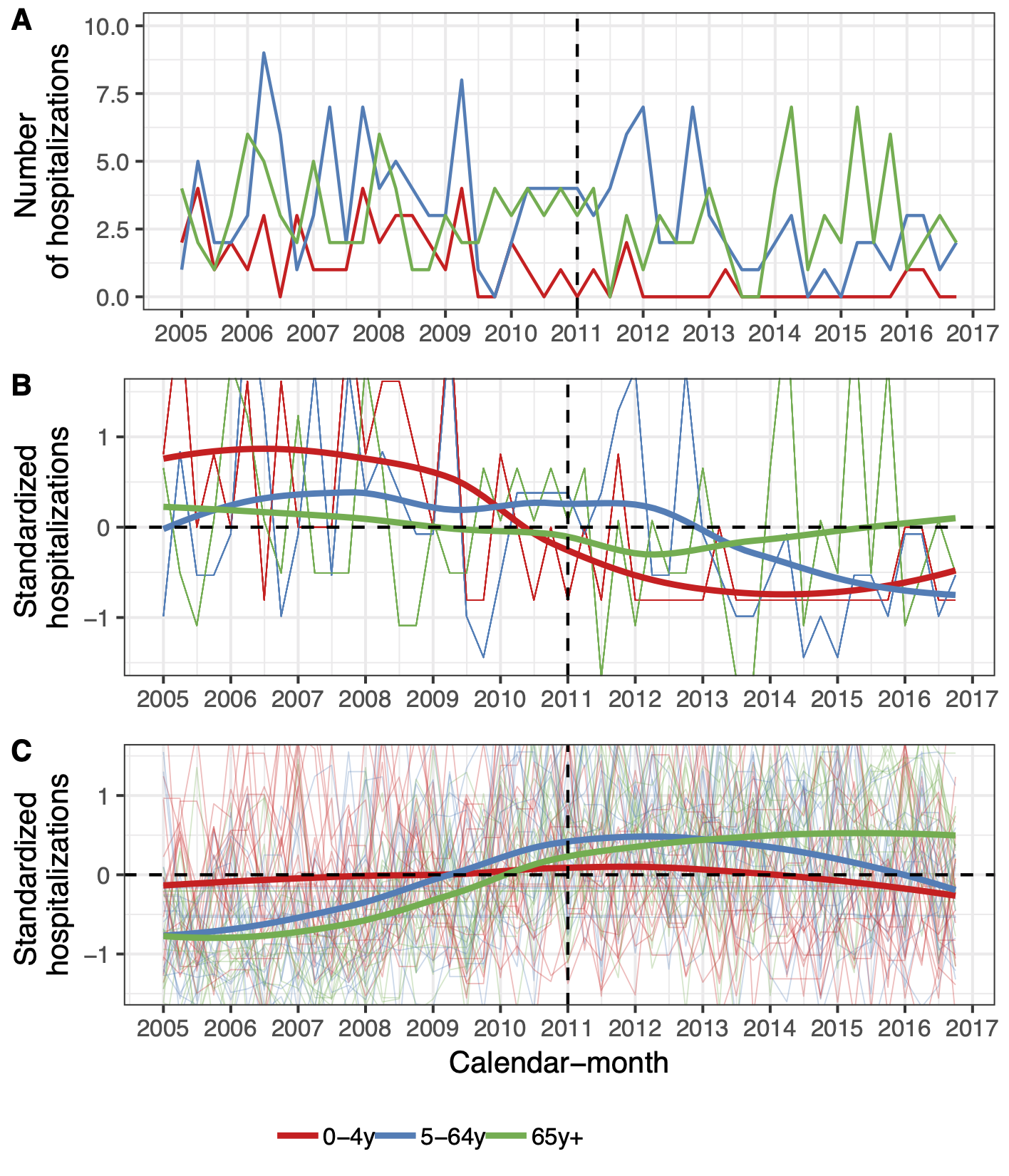

Supplement: S13 Fig — The population is divided into three age-groups, listed in the figure’s legend. Panel A shows the absolute quarterly number of hospital admissions due to invasive pneumococcal disease (IPD), regardless of serotype. Panels B and C, depict the standardized quarterly number of IPD hospitalizations (Panel B) and all-cause hospitalizations (Panel C) per age-group. The Y-axis represents the number of standard deviations from the mean hospitalizations for each quarter and each age-group. The horizontal dotted lines represent values that are zero standard deviations from the mean and the vertical dotted lines represent the start of the vaccine intervention. Locally estimated scatter-plot smoothing (LOESS) produced an average trend. Panels B and C have been magnified to emphasize the interpretation of the trend line. Panels B and C show that standardized hospitalizations for IPD decreased in all age-groups, relative to the standardized hospitalizations, regardless of cause. (TIF) [file pone.0249497.s014.tif]

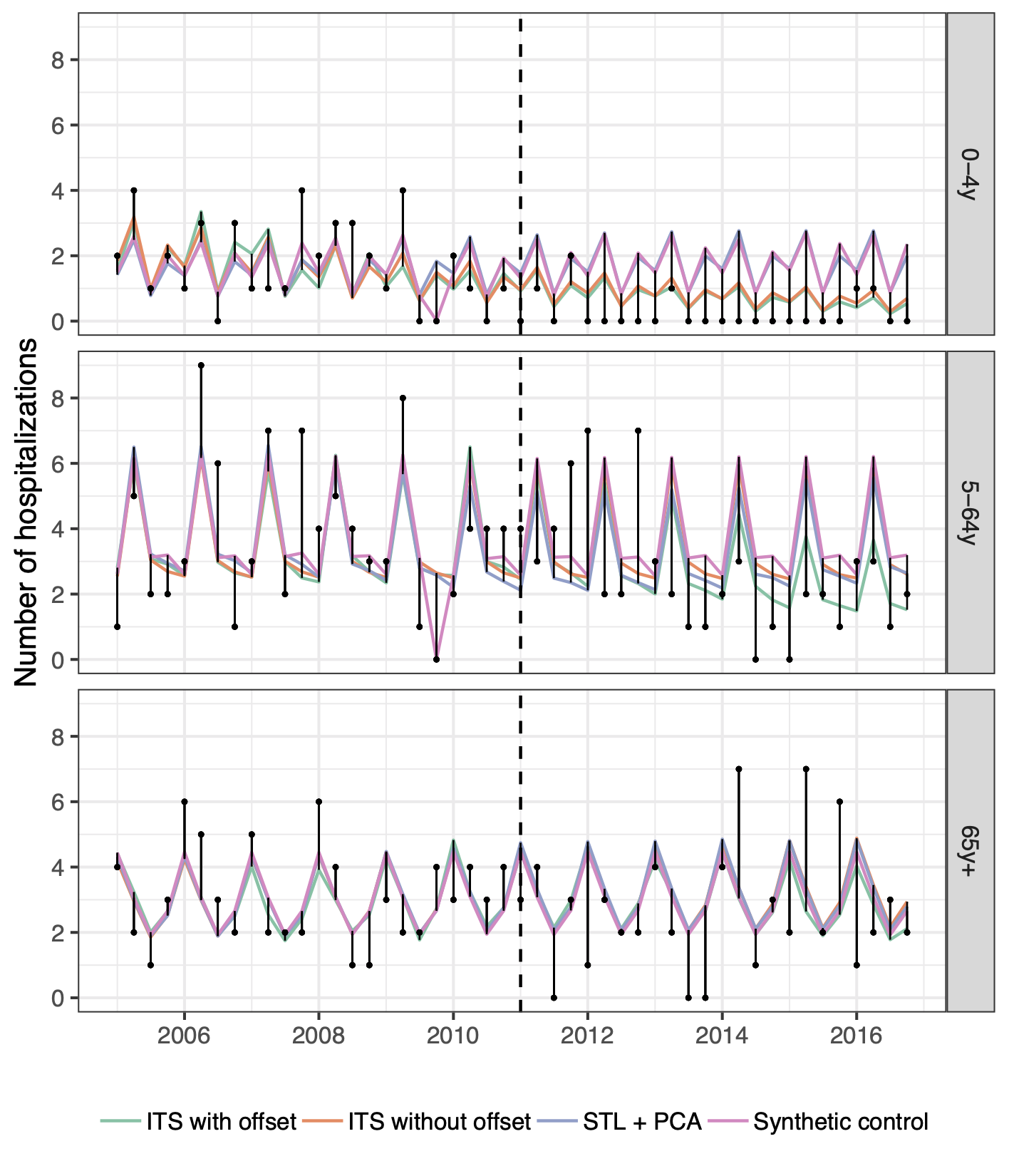

Supplement: S14 Fig — Observed cases are illustrated as black points, and predicted number of cases are drawn as lines for each of the component models. The start of the vaccine period is delineated with a vertical black dotted line. Each component model was fitted to the observed number of cases in the pre-vaccine period. They were then used to predict the number of cases that would have occurred in the post-vaccine period, had the vaccine not been introduced. The distance between the observed and predicted cases for each year-quarter is depicted with a thin black line. Longer distances suggest a larger discrepancy between observed and predicted cases. (TIF) [file pone.0249497.s015.tif]

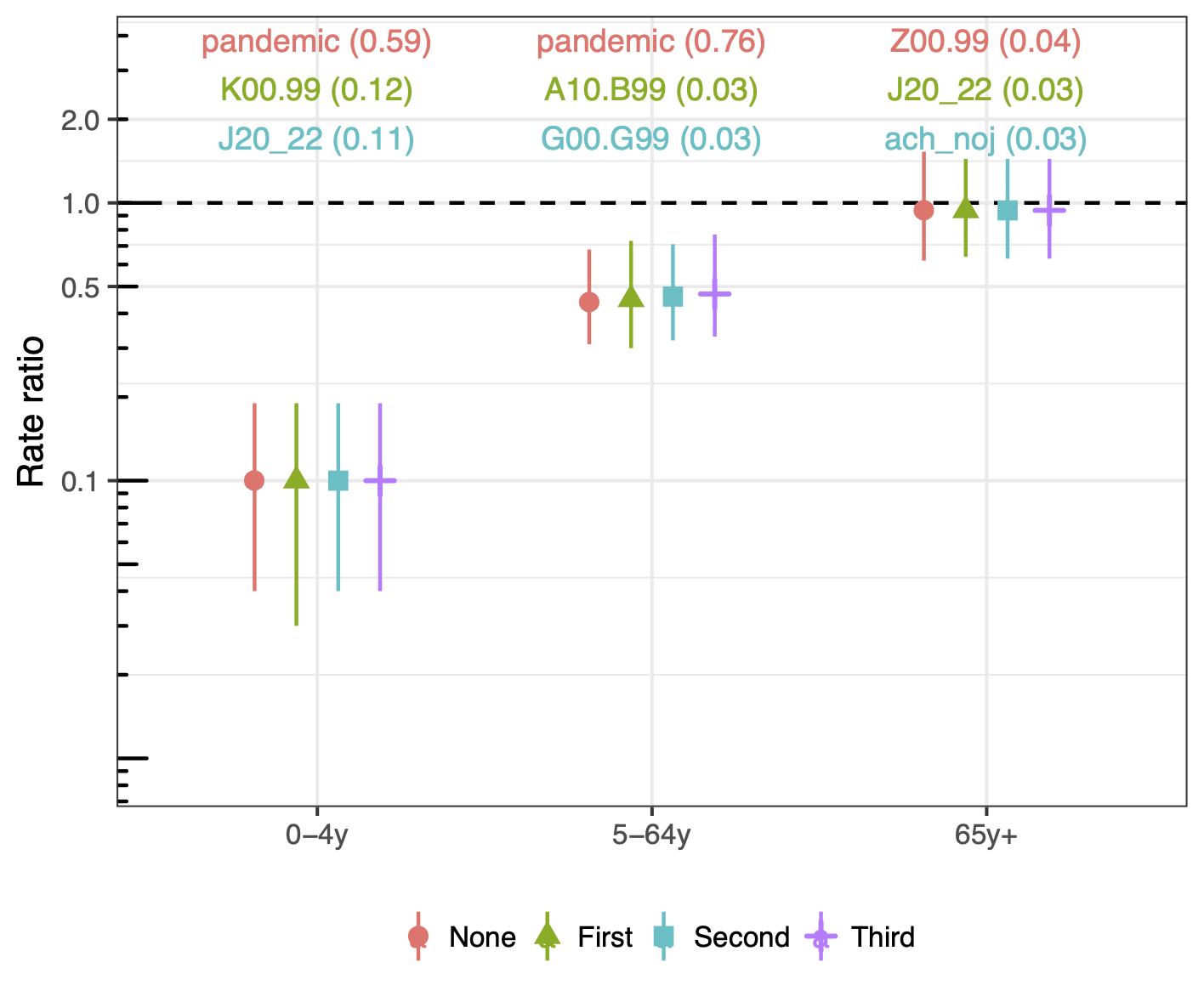

Supplement: S15 Fig — The leftmost point and confidence interval represents the full synthetic model used in the analysis. The same colored label shows the top control and its associated inclusion probability in the Bayesian variable selection process. From left to right, the top control is removed, the model is refitted on the remaining controls and the corresponding rate ratio illustrated with a point and interval. The results are largely invariant to the controls used. (TIF) [file pone.0249497.s016.tif]

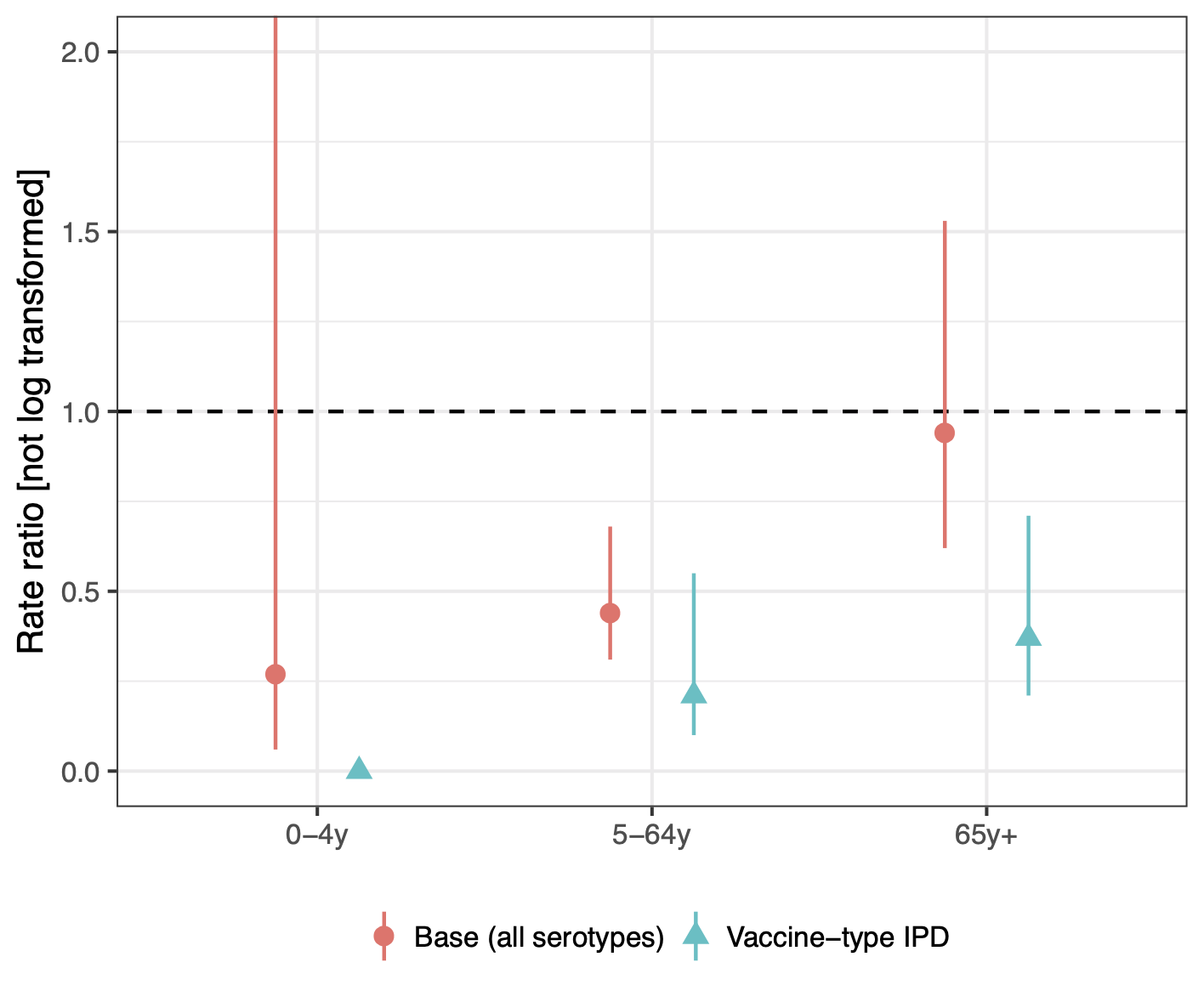

Supplement: S16 Fig — Culture- or PCR-confirmed IPD regardless of serotype, the case-definition used in the main analysis, is shown with a red point and intervals. The green point represents vaccine-type IPD. The number of nonvaccine-type IPD in the pre-vaccine period was not large enough to fit any of the time series models. The figure shows that the impact of the 10-valent pneumococcal Haemophilus influenzae Protein D conjugate vaccine (PHiD-CV) on vaccine-type is considerable in all age-groups. (TIF) [file pone.0249497.s017.tif]
