# Supplementary material for: The impact and cost-effectiveness of introducing the 10-valent pneumococcal conjugate vaccine into the paediatric immunisation programme in Iceland—A population-based time series analysis
Source: PLoS One. 2021 Apr 8;16(4):e0249497. doi: 10.1371/journal.pone.0249497 (PMC8031404; doi:10.1371/journal.pone.0249497)
Supplement: S1 Table — (DOCX) [file pone.0249497.s018.docx]

**S1 Table. The weights used to produce the final stacked model from the component models.**

| Disease category | Age-group | Synthetic controls | ITS with offset | ITS without offset | STL + PCA |
| --- | --- | --- | --- | --- | --- |
| AOM visits | 0y | 0.221 | 0.000 | 0.121 | 0.659 |
| AOM visits | 1y | 0.149 | 0.000 | 0.610 | 0.241 |
| AOM visits | 2y | 0.000 | 0.000 | 0.479 | 0.521 |
| AOM visits | 3-4y | 0.661 | 0.000 | 0.339 | 0.000 |
| AOM visits | 5-9y | 0.726 | 0.000 | 0.274 | 0.000 |
| AOM visits | 10-14y | 1.000 | 0.000 | 0.000 | 0.000 |
| AOM visits | 15-19y | 0.018 | 0.000 | 0.078 | 0.904 |
| Pneumonia hospitalizations | 0-4y | 0.912 | 0.001 | 0.087 | 0.000 |
| Pneumonia hospitalizations | 5-19y | 1.000 | 0.000 | 0.000 | 0.000 |
| Pneumonia hospitalizations | 20-39y | 0.246 | 0.124 | 0.000 | 0.629 |
| Pneumonia hospitalizations | 40-64y | 0.241 | 0.000 | 0.000 | 0.759 |
| Pneumonia hospitalizations | 65-79y | 0.000 | 0.934 | 0.066 | 0.000 |
| Pneumonia hospitalizations | 80+ | 0.000 | 0.472 | 0.528 | 0.000 |
| IPD hospitalizations | 0-4y | 0.001 | 0.999 | 0.000 | 0.000 |
| IPD hospitalizations | 5-64y | 1.000 | 0.000 | 0.000 | 0.000 |
| IPD hospitalizations | 65y+ | 1.000 | 0.000 | 0.000 | 0.000 |

The weights for each component model were obtained by minimizing the leave-one-out mean squared error.
